# Exploiting recognition-mediated assembly and reactivity in [2]rotaxane formation†
†Electronic supplementary information (ESI) available: Details of kinetic experiments, computational methods and characterisation data for all compounds are provided. See DOI: 10.1039/c5sc04805b


**DOI:** 10.1039/c5sc04805b

**Published:** 2016-01-15

**Authors:** Annick Vidonne, Tamara Kosikova, Douglas Philp

**Affiliations:** a School of Chemistry and EaStCHEM , University of St Andrews , North Haugh St Andrews , Fife KY16 9ST , UK . Email: d.philp@st-andrews.ac.uk ; Fax: +44 (0)1334 463808 ; Tel: +44 (0)1334 467264

## Abstract

A small molecular reaction network exploits recognition-mediated reactive processes in order to drive the assembly and formation of both a self-replicating linear template (thread) and a [2]rotaxane, in which the linear template is encircled by a diamide macrocycle. Complementary recognition sites, placed at strategic positions on the reactive building blocks, drive these assembly and replication processes. Template-instructed experiments show that the thread is capable of efficient self-replication and that no cross-catalytic relationships exist between the thread and the [2]rotaxane. The rate of [2]rotaxane formation is insensitive to the addition of a preformed template, however, [2]rotaxane formation does show enhanced diastereoselectivity, most likely originating from its recognition-mediated formation through a ternary reactive complex.

## Abstract 2

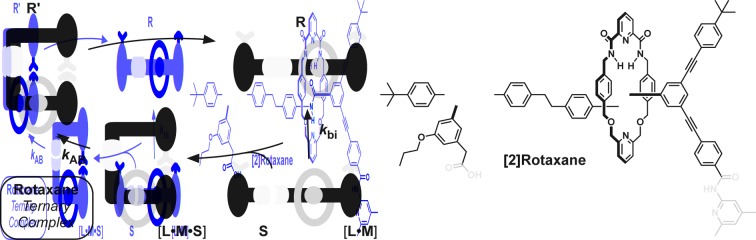
A ternary complex facilitates the recognition-mediated formation of a [2]rotaxane.

## Introduction

In recent years, advances in supramolecular synthesis have permitted the construction of a diverse array of molecular machinery^1^ and nanometre-scale^2^ objects. In general, the approaches used in these construction processes have featured sophisticated algorithmic self-organisation^3^ and self-assembly^4^ processes to control the precise arrangement of structural subunits in space. Despite the attractions of self-assembly processes, other construction methods have also been discussed. In particular, Drexler highlighted^5^ a radical alternative – the fabrication of replicable networks *via* atom-by-atom engineering. Whilst this proposal as described^6^ may be implausible, replication processes remain an attractive alternative to the traditional synthetic approaches. In the last 20 years, numerous examples of synthetic replicating systems capable of templating and catalysing their own synthesis have appeared^7^ in the chemical literature.

Self-replication^8^ is a subset of autocatalytic reactions.^9^ In an autocatalytic reaction, a product formed during the reaction acts as a catalyst for its own formation. A system in which self-replication is operating can be defined as an autocatalytic reaction capable of transmitting (and amplifying) structural information. All the synthetic self-replicating systems reported to date utilise precursor subunits that incorporate mutually complementary recognition elements and reactive functionalities. These features have been combined to engineer linear self-complementary platforms capable of autocatalytic self-propagation.

**Fig. 1:**
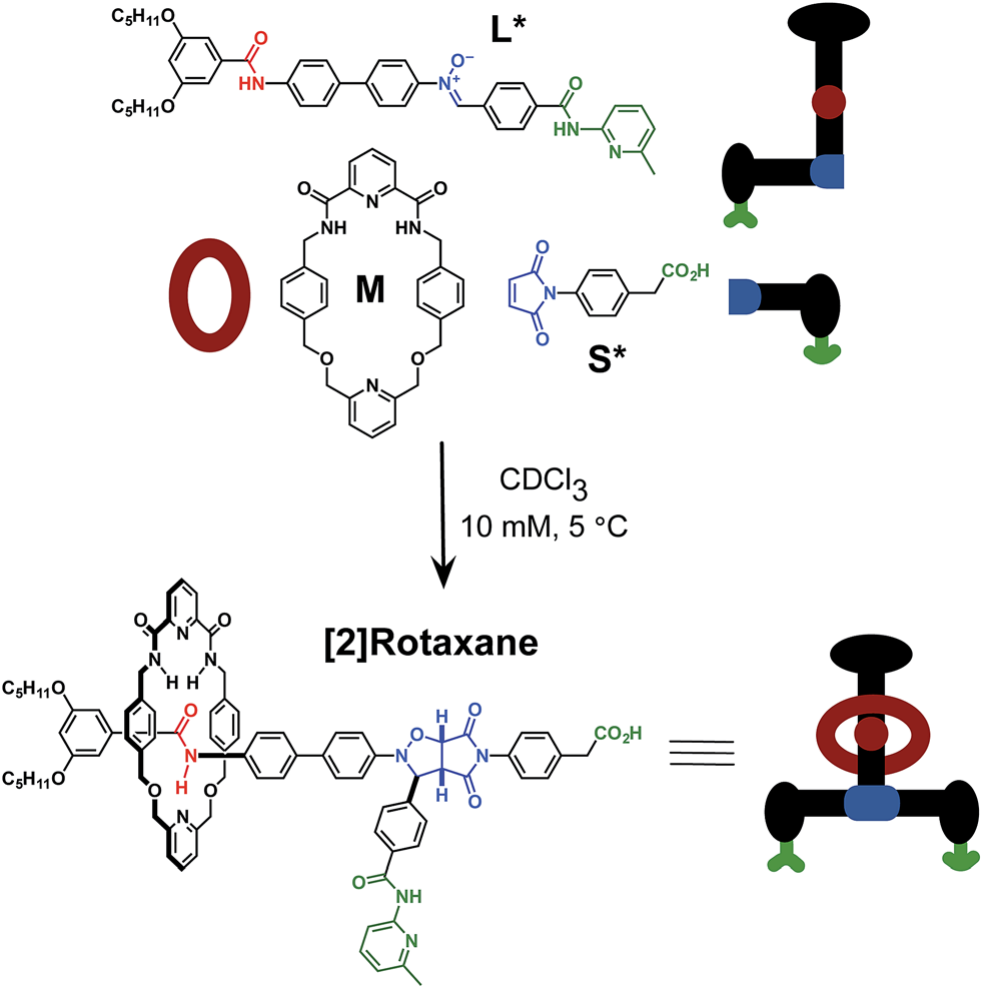
Cartoon and chemical structure representations of a kinetic framework utilising orthogonal recognition processes to drive^11^ the assembly and replication of a [2]rotaxane. Green represents the recognition elements required for self-replication; blue represents the reactive components and red highlights the macrocycle-binding site required for rotaxane assembly.

Rotaxanes are archetypal examples of interlocked molecular architectures;^10^ they consist of a linear molecule, known as the thread, that is encircled by a macrocycle and end-groups (stoppers) large enough to prevent dethreading. Recently, we described^11^ a kinetics framework for embedding the synthesis of a [2]rotaxane within a self-replicating network. This strategy (Fig. 1) was based on incorporating an efficient replicating template7d,^12^ as one of the stoppers on the rotaxane. Whilst this approach was successful in assembling and replicating the [2]rotaxane, it suffers from a number of shortcomings.

Firstly, since the stopper is at the periphery of the structure (Fig. 1), the presence or absence of the macrocycle, in principle, has little effect on the replication process. This shortcoming manifests itself as strong crosscatalytic relationships within the reaction network. In fact, in this system, the [2]rotaxane is an equally good catalyst for the formation of the thread as the thread is for its own formation. This crosscatalysis introduces an inherent limitation to the achievable thread : [2]rotaxane ratio.

More seriously, there are several unproductive binding modes available to the macrocycle within this design that prevent the optimal replication of the [2]rotaxane. In particular, linear component L* contains the diaryl amide binding site for the macrocycle (Fig. 1, red), the nitrone (Fig. 1, blue) that reacts with maleimide S* to form the replicating framework and the amidopyridine recognition element (Fig. 1, green). In order to ensure optimal replication, macrocycle **M**^11^,^13^ must bind only to the diaryl amide recognition site. In practice, however, there are significant populations of complexes where the macrocycle is bound either to the nitrone or the amidopyridine present in L*. These unproductive binding modes reduce the replication efficiency by around 75%.

**Fig. 2:**
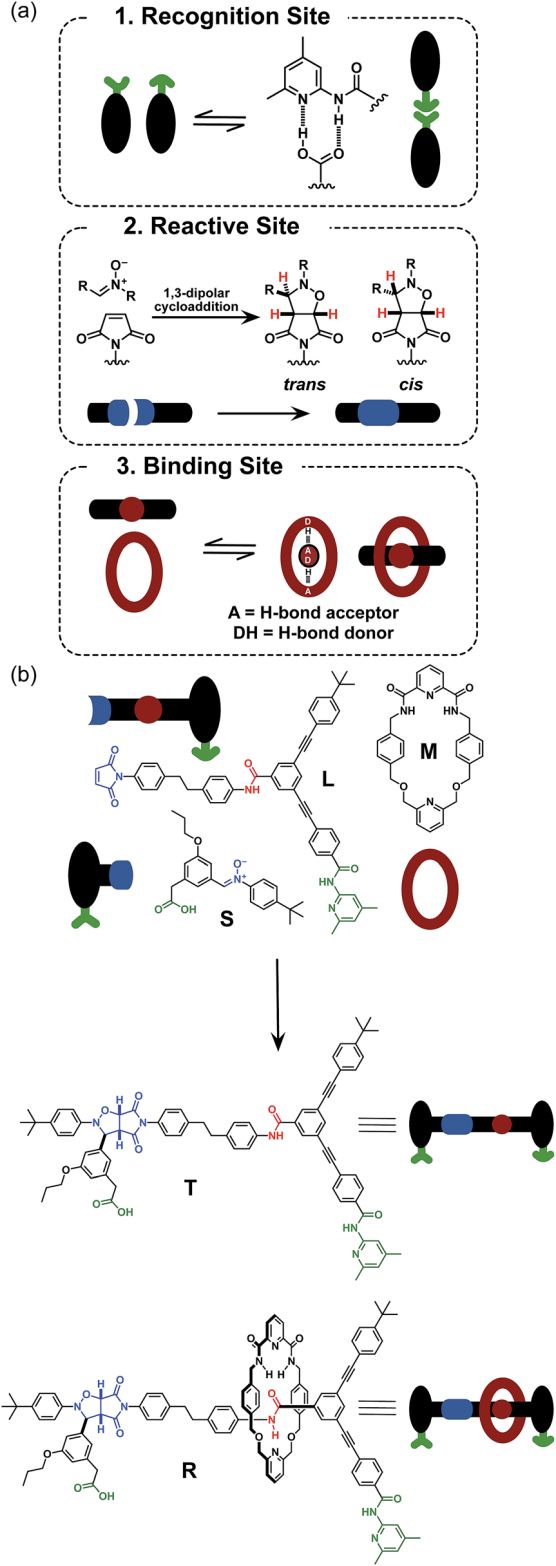
(a) Cartoon representation of the recognition, reactive and macrocycle-binding elements required for the integration of the rotaxane assembly with replication. (b) Cartoon and chemical structure representation of the components employed in the recognition-mediated rotaxane assembly. Green represents the recognition elements required for self-replication; blue represents the reactive components and red highlights the macrocycle-binding site.

Here, we report an alternative strategy^14^ for the integration of rotaxane assembly and replication. This approach (Fig. 2) involves the isolation of all of the recognition events required for the assembly and replication from each other at specific loci within the molecular structures of the [2]rotaxane components. In order to achieve this isolation, one of the recognition elements required to drive the replication must be placed on each of the two stoppers (Fig. 2a). This design change permits the use of the same 1,3-dipolar cycloaddition between a nitrone and a maleimide to connect the maleimide **L** and nitrone stopper **S** components together. This reaction affords two pairs of diastereoisomeric cycloadducts (Fig. 2a) – labelled7d,^15^ *trans* and *cis*, and the reaction is rather insensitive to electronic effects, usually giving a *trans*/*cis* ratio between 2.7 : 1 and 3.3 : 1. We have described7d,^11^,^12^,^14^,^15^ the use of this reaction in a number of successful recognition-mediated reaction systems. The same macrocycle and diaryl amide binding motif, used^11^ in our previous design, can also be utilised in this context. These design changes lead, ultimately, to the identification of the target thread **T** and [2]rotaxane **R** (Fig. 2b). Thread **T** and rotaxane **R** can be constructed from the corresponding maleimide **L**, nitrone **S** and, for the rotaxane only, macrocycle **M** (Fig. 2b). This new design removes any possibility of unproductive binding from the system. Macrocycle **M** can associate only with the target diaryl amide (Fig. 2b, red) present in **L**. The two methyl groups present on the amidopyridine ring in **L** (Fig. 2b, green) prevent the association of **M** with this unit. Similarly, the *n*-propyloxy and *tert*-butyl groups present in **S** prevent the association of the macrocycle with the nitrone.

## Results and discussion

The synthesis of maleimide **L**, incorporating the maleimide reactive site, the amidopyridine recognition site and the amide-binding site was accomplished^14^ in five steps starting from methyl 3-bromo-5-iodobenzoate (for details, see ESI†). Compound **S**, incorporating the nitrone reactive site and the carboxylic acid recognition site, and its seven-step synthesis, as well as the synthesis of macrocycle **M**, have been reported^11^,^13^,^14^ previously.

**Fig. 3:**
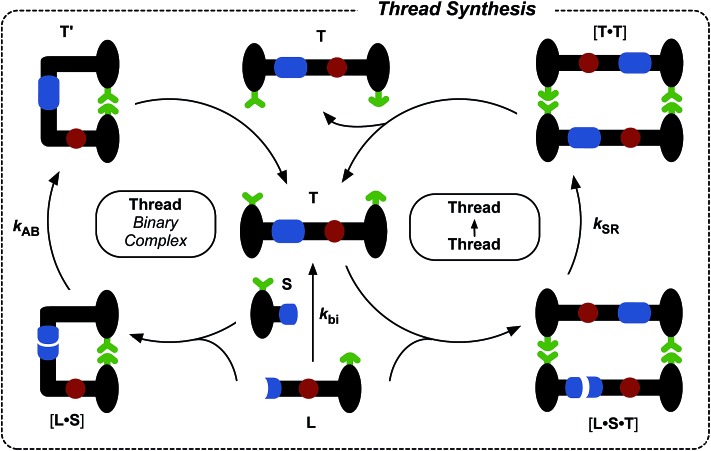
Schematic representation of the three pathways leading to thread **T** formation. Namely, **T** can be formed through the bimolecular reaction of maleimide **L** with stopper **S**. These two components can also associate *via* the recognition elements (green) to give a binary reactive complex [**L**·**S**] which brings the reactive components (blue) into a reactive conformation, affording closed thread **T′**, which is in equilibrium with its open form **T**. The open **T** can associate with the unreacted **L** and **S** in a catalytically-active ternary complex [**T**·**L**·**S**], which reacts to form template duplex [**T**·**T**], the dissociation of which fulfils the autocatalytic pathway.

Initially, we wished to establish that thread **T** was capable of replication in isolation from the macrocycle. Therefore, we undertook a kinetic analysis of the reactions involving the thread only, shown in Fig. 3. In principle, thread **T** can be formed through three separate pathways. The first pathway is the uncatalysed bimolecular reaction between maleimide **L** and nitrone **S** to form **T** (Fig. 3, lower centre). Two recognition-mediated pathways can also lead to the formation of thread **T**. Firstly, the association of maleimide **L** with nitrone **S** through their mutually complementary recognition sites results in the formation of the binary complex [**L**·**S**]. The reaction between **L** and **S** within this complex is pseudounimolecular and affords the closed template **T′**. This structure is, in principle, in equilibrium with the open thread **T** (Fig. 3, top left). Secondly, open thread **T** can assemble **L** and **S** to form the ternary complex [**L**·**S**·**T**] within which the reaction between **L** and **S** is also pseudounimolecular (Fig. 3, right). The resulting product duplex [**T**·**T**] is capable of dissociating to return two molecules of thread to the autocatalytic cycle, thus completing the replication process.

In order to determine which of these recognition-mediated pathways are active in this system, we performed a series of kinetic experiments. A solution of maleimide **L** and nitrone **S**, with starting concentrations of the two reagents of 20 mM, was prepared in CDCl_3_. This concentration was chosen for all experiments reported here as it provides a compromise between a number of competing factors. There is a complex interplay between the various binding equilibria illustrated in Fig. 3 and the rates of the recognition-mediated reactions. It is crucial to balance the *K*_d_ (4 mM) for the single point associations, *e.g.***L** + **S** → [**L**·**S**], with the stability of the ternary complex [**L**·**S**·**T**]. The reaction should be performed at a concentration above the *K*_d_ for the single point association, but not so high that the binary complex [**L**·**S**] is dominant in the system. In general, there is an optimum concentration window7*d* in which replication can be observed, and we estimated that a concentration of 20 mM would be close to the centre of this window for this system.

**Fig. 4:**
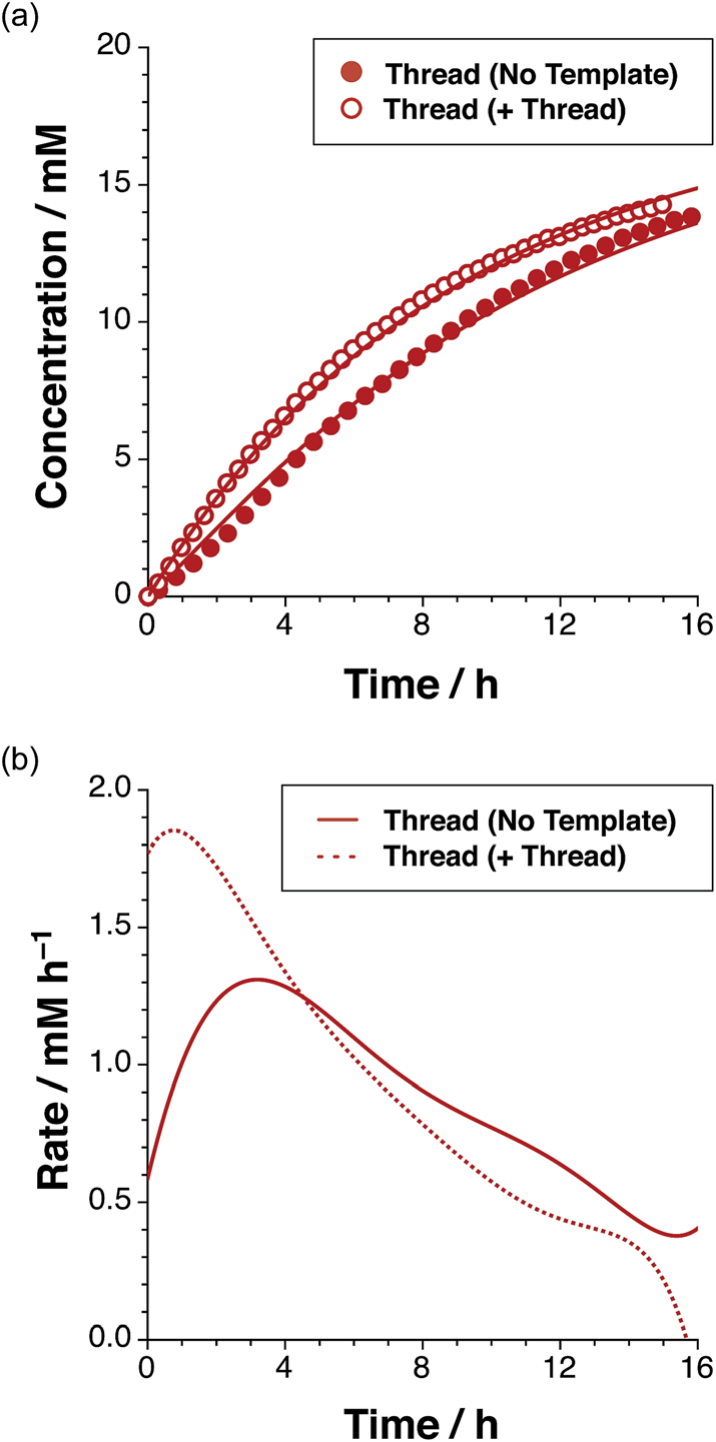
(a) Concentration *vs.* time profile and (b) rate *vs.* time profile for the reaction of maleimide **L** with stopper **S**, to give thread **T** in the absence of any added template (filled red circles/full red line) and in the presence of 38 mol% pre-formed **T** (empty red circles/dashed red line), added at *t* = 0 as determined using 500 MHz ^1^H NMR spectroscopy (15 °C, CDCl_3_). Formation of *cis*-**T** is not plotted for clarity (concentration less than 0.6 mM after 15 h).

The time course of the reaction (Fig. 4a, filled red circles) at 15 °C was evaluated using 500 MHz ^1^H NMR spectroscopy. The disappearance of the resonance arising from the maleimide protons present in **L** at *δ* 6.85 and the simultaneous appearance of the resonances corresponding to *trans*-**T**, initially at *δ* 5.78, were monitored (for details, see ESI†). The formation of **T** is very diastereoselective, therefore quantification of the small amount of *cis*-**T**, characterised by the resonances around *δ* 4.76, was challenging. Therefore, the concentration–time profiles presented here show only the data for *trans*-**T**.

After 15 hours, the reaction (Fig. 4a) between maleimide **L** and nitrone **S** reached 67% overall conversion and the diastereoselectivity was excellent – 20 : 1 in favour of *trans*-**T**. The formation of the major isoxazolidine product, *trans*-**T**, displays a rate^16^ maximum (1.31 mM h^–1^ at *t* = 3.2 h), characteristic of a self-replicating system. In order to confirm the ability of *trans*-**T** to template its own formation, *i.e.* to replicate, a further set of experiments were conducted. A solution of maleimide **L** and nitrone **S** with starting concentrations of the two reagents of 20 mM, was prepared in CDCl_3_. Pre-synthesised *trans*-**T** (38 mol%) was added to this solution and the time course of this reaction at 15 °C was, once again, evaluated using 500 MHz ^1^H NMR spectroscopy (Fig. 4a). In this case, an increase in the initial rate of formation of *trans*-**T** (71% conversion after 15 h, with 20 : 1 *trans* : *cis* ratio), but not of *cis*-**T**, was observed and the rate–time profile displays a rate maximum (1.85 mM h^–1^) at *t* = 0.75 h (Fig. 4b). This experiment establishes unambiguously that *trans*-**T** acts as a template for its own formation, *i.e.* it is capable of replication.

By contrast, in the reaction between maleimide **L** and a recognition-disabled nitrone stopper (for details, see ESI†), which does not possess the carboxylic acid recognition site, the overall conversion after 15 hours was only 37% for *trans*-**T** and 16% for *cis*-**T**, and the diastereoselectivity was only 2.3 : 1 in favour of the *trans* isoxazolidine. The reliance of the system on molecular recognition was further demonstrated by examining the formation of **T** in the presence of an inhibitor (for details, see ESI†) – 2.6 equivalents of 4-bromophenylacetic acid. This compound, although unreactive, is still capable of binding to the amidopyridine recognition sites present in **L** and **T**, thereby interfering with the recognition processes critical to the operation of the autocatalytic cycle. In this experiment, a decrease in the rate of the reaction, conversion to *trans*-**T** reached only 36% after 15 h, was observed and the ratio of *trans*-**T** to *cis*-**T** was 8.9 : 1 after 15 h. The conversion to *trans*-**T** observed in the inhibitor experiment is nearly identical to the conversion observed in the kinetic experiment where the thread formation can proceed only through the bimolecular pathway (37% after 15 h). This decrease in conversion was accompanied by the disappearance of the sigmoidal concentration–time profile for *trans*-**T**. Taken together, these kinetic experiments clearly demonstrate that the formation of *trans*-**T** is the result of a recognition-mediated replication process.

**Fig. 5:**
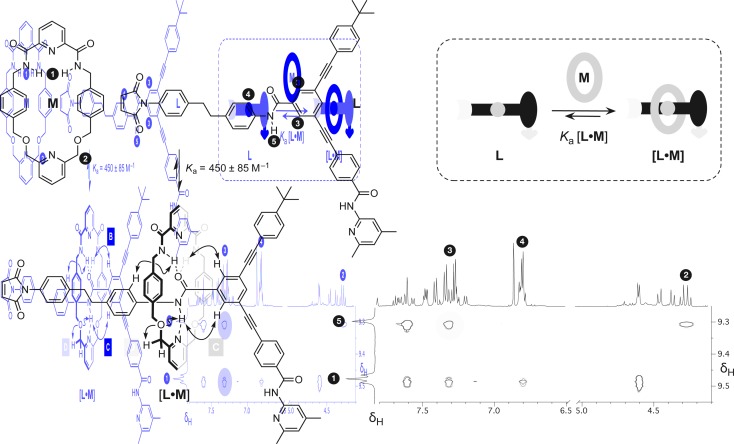
Binding study examining the formation of pseudorotaxane [**L**·**M**] from the individual components **L** and **M** (25 mM) in CDCl_3_ at 0 °C. Partial ^1^H–^1^H ROESY NMR (499.9 MHz) correlation spectrum highlights the ROE cross peaks showing through space interactions between the maleimide component and the macrocycle. Unassigned cross peaks represent the through space interactions within the individual components. Cartoon representation shows the association of the macrocycle with the diaryl amide-binding site (red) on the maleimide component.

Having established the replicating ability of the core thread structure, we next sought to establish the viability of the linear component **L** as a suitable host for macrocycle **M**. The association of the macrocycle to the amide binding site is a key feature of the rotaxane. The position of the equilibrium between the linear component **L** and macrocycle **M** (Fig. 5, cartoon) and the corresponding [**L**·**M**] complex is crucial to the success of the formation of the [2]rotaxane. The ability of maleimide **L** to thread through the cavity of macrocycle **M** and to associate with the diaryl amide was assessed by performing a binding experiment. A solution containing a 1 : 1 ratio of macrocycle **M** and maleimide **L** (25 mM each) in CDCl_3_ was prepared and analysed at 0 °C using ^1^H NMR spectroscopy. The 499.9 MHz ^1^H NMR spectrum of this mixture (for details, see ESI†) exhibits three different sets of resonances, corresponding to (i) free **M**, (ii) uncomplexed **L**, and (iii) the complex [**L**·**M**]. This observation indicates that the bound and unbound species equilibrate slowly with one another on the ^1^H NMR chemical shift timescale. This phenomenon allowed the determination of the association constant using^17^ the single-point method and a value for *K*_a_ was estimated to be 450 ± 85 M^–1^ in CDCl_3_ at 0 °C. The chemical shift changes observed are entirely consistent with pseudorotaxane formation. The resonance arising from the macrocycle NH protons (Fig. 5, protons marked 1) is shifted downfield (+1.37 ppm) as a result of the hydrogen bonding between these protons and the maleimide amide carbonyl group. The upfield shifts for the resonances of the macrocycle phenylene protons are characteristic of protons residing in the shielding zone of an aromatic ring. The downfield resonance arising from the NH proton associated with the amide in **L**, which is the binding target of macrocycle **M**, (Fig. 5, proton marked 5, *δ* 9.29) suggests that this proton is hydrogen bonded to the macrocycle pyridine nitrogen atom. Additionally, a complex pattern resulting from the resonances of the macrocycle methylene groups was observed; the end-to-end asymmetry of **L** renders the protons located on opposite faces of the macrocycle diastereotopic. A ^1^H–^1^H ROESY NMR experiment (Fig. 5) gave detailed structural information about the [**L**·**M**] complex and the ROE cross peaks observed prove unambiguously that pseudorotaxane [**L**·**M**] has the expected geometry.

**Fig. 6:**
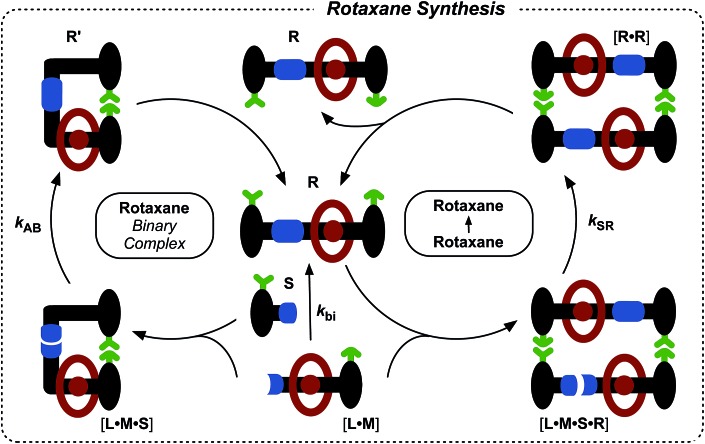
Schematic representation of the three pathways leading to rotaxane **R** formation. Specifically, **R** can be formed through the bimolecular reaction of pseudorotaxane complex [**L**·**M**] with stopper **S**. The pseudorotaxane can also associate with **S***via* the recognition elements (green) to give a ternary reactive complex [**L**·**M**·**S**] which brings the reactive components (blue) into a reactive conformation, affording closed thread **R′**, which is in equilibrium with its open form **R**. The open **R** can associate with the unreacted [**L**·**M**] and **S** in a catalytically-active quaternary complex [**R**·**L**·**M**·**S**], which reacts to form template duplex [**R**·**R**], the dissociation of which fulfils the autocatalytic pathway.

Having established that the formation of the desired pseudorotaxane complex is possible, we wished to examine the formation of the rotaxane from the individual building blocks (Fig. 6). As with the formation of **T**, there are three main pathways that can afford rotaxane **R** within this kinetic framework. The bimolecular reaction between pseudorotaxane [**L**·**M**] (Fig. 6, centre) and stoppering reagent **S** affords the [2]rotaxane **R**. Stoppering reagent **S** and maleimide **L** bear complementary recognition sites on their stoppers, hence **S** and [**L**·**M**] can bind reversibly to form a reactive ternary complex [**S**·**L**·**M**], which results in the formation of closed rotaxane **R′** (Fig. 6, left) through a recognition-mediated reaction. The closed form **R′** is in equilibrium with the open rotaxane template **R** (Fig. 6, top left), which can also associate with the pseudorotaxane and stopper in a catalytic quaternary complex [**L**·**M**·**S**·**R**] (Fig. 6, right). Bond formation occurs between **S** and [**L**·**M**] to give the product duplex [**R**·**R**], which then dissociates to return two molecules of **R** to the start of the autocatalytic cycle (Fig. 6, right).

In order to ensure that rotaxane **R** can be formed using our three building blocks, its synthesis was attempted on a preparative scale. A mixture of maleimide **L** and macrocycle **M** was pre-equilibrated in chloroform at ambient temperature in order to allow the formation of the pseudorotaxane. The addition of nitrone **S** to this solution afforded, after six days at ambient temperature, rotaxane **R** in 46% yield, as a 18 : 1 mixture of its *trans*- and *cis*-diastereoisomers, and thread **T** in 45% yield, as a 35 : 1 mixture of its *trans*- and *cis*-diastereoisomers after purification using column chromatography. These products were characterised using a combination of one- and two-dimensional NMR experiments (see ESI†).

**Fig. 7:**
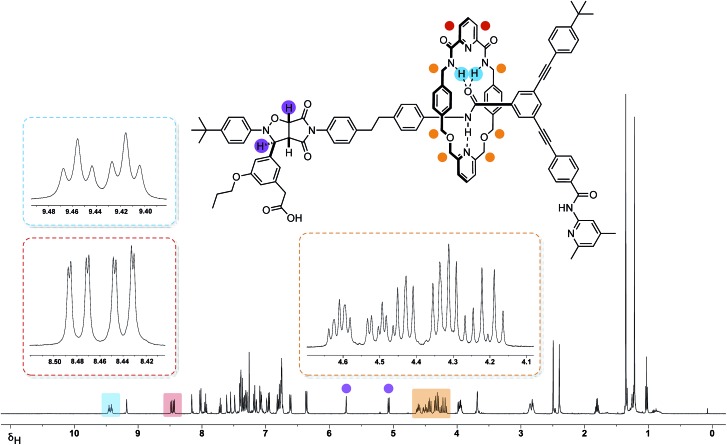
^1^H NMR spectrum (499.9 MHz, 293 K, CDCl_3_) of rotaxane *trans*-**R** together with selected proton assignments. Resonances arising from the protons on the isoxazolidine ring are highlighted in purple. Resonances arising from the magnetically-inequivalent pyridine protons (red), NH (blue) and methylene protons (orange) located on the macrocycle, are shown as partial inset spectra. The sample also contains around 5% of *cis*-**R**.

The 499.9 MHz ^1^H NMR spectrum of *trans*-**R** (Fig. 7) reveals a number of features consistent with the mechanical interlocking of the thread and macrocycle components. The resonances arising from the macrocycle NH protons are shifted significantly downfield (*δ* 9.46 and 9.42, Δ*δ* –1.58 and –1.54) and appear (Fig. 7, blue) as two separate triplets. The observation of two resonances for these protons is consistent with the reduction in symmetry of the macrocycle from *C*_S_ to *C*_1_ on the formation of the cycloadduct. This effect also manifests itself in the observation of two magnetically inequivalent pyridine ring protons (Fig. 7, red). The magnitudes of these chemical shift differences are surprising given the somewhat remote relative locations of the macrocycle and cycloadduct, but may reflect the fact that, in solution, the closed structure **R′** (Fig. 6, top left) predominates. The end-to-end asymmetry that arises from the threading of the linear component through the macrocycle is maintained in the rotaxane and is responsible for the complex pattern observed (Fig. 7, orange) for the resonances arising from the CH_2_ groups present in the macrocycle.

**Fig. 8:**
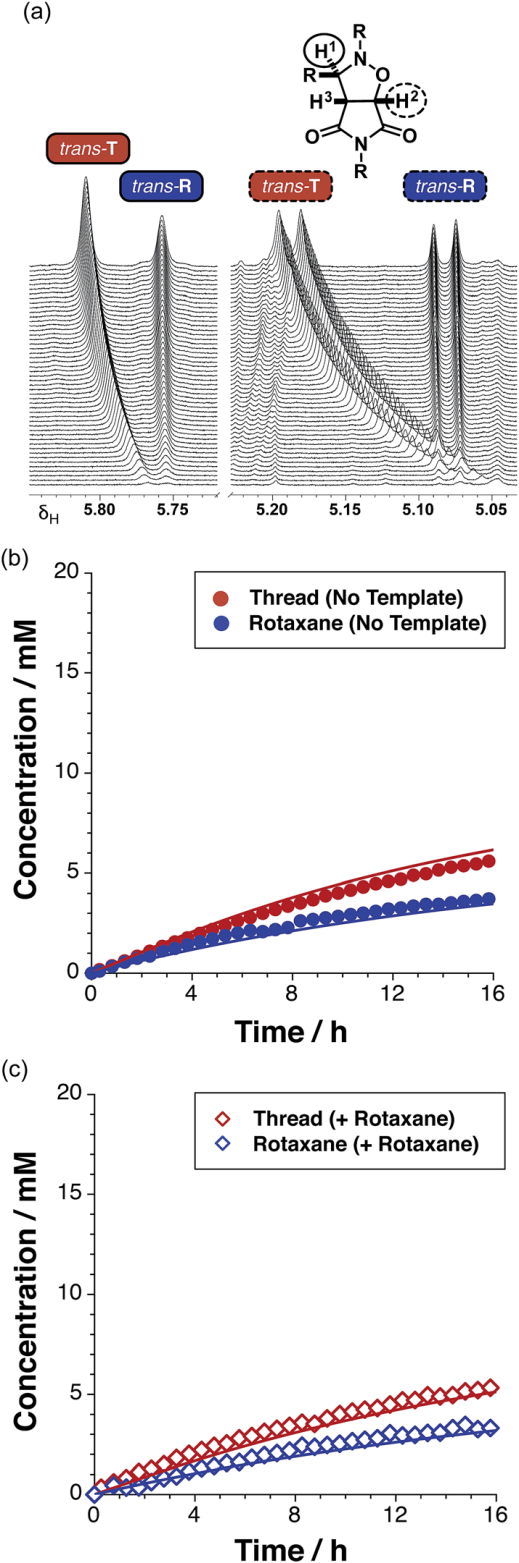
(a) Arrayed partial 500.1 MHz ^1^H NMR spectra recorded during the kinetic experiment for the reaction between maleimide **L** and nitrone **S** in the presence of macrocycle **M**, showing the appearance of resonances associated with protons H^1^ (full line) and H^2^ (dashed line) on the *trans*-isoxazolidine ring of **T** (red) and **R** (blue). This NMR data can be converted into the corresponding (b) concentration *vs.* time profile for this reaction – *trans*-**T** (filled red circles) and *trans*-**R** (filled blue circles). (c) Concentration *vs.* time profile for the reaction of maleimide **L** with stopper **S** and macrocycle **M** in the presence of 38 mol% pre-formed *trans*-**R** added at *t* = 0, to give the thread **T** (empty red diamonds) and rotaxane **R** (empty blue diamonds). Concentrations were determined using 500.1 MHz ^1^H NMR spectroscopy (15 °C, CDCl_3_).

Having established that the rotaxane synthesis is viable, we next undertook a kinetic analysis aimed at assessing the relative efficiencies of the different reaction pathways available for the formation of the rotaxane. To this end, we first explored the formation of **R** in the absence of any preformed template. An equimolar solution of maleimide **L** and macrocycle **M** in CDCl_3_ was pre-equilibrated, before the addition of nitrone **S** to the mixture. The starting concentration of the three reagents was 19 mM. The time course of the reaction was evaluated using 500.1 MHz ^1^H NMR spectroscopy. The appearance (Fig. 8a) of the resonance that arises from *trans*-**T** (initially at *δ* 5.77), and of a resonance that arises from *trans*-**R** (*δ* 5.76), was monitored. Interestingly, while the resonance that arises from *trans*-**T** experiences a downfield shift from *δ* 5.77 to *δ* 5.81, the corresponding resonance arising from *trans*-**R** does not exhibit any chemical shift change. A similar pattern of shift changes is also observed for the resonance that arises from *trans*-**T** initially at *δ* 5.06, which experiences a downfield shift to *δ* 5.19 over the course of the reaction. However, the resonance that arises from *trans*-**R** (*δ* 5.08), once again, does not exhibit any chemical shift change. The spectroscopic behaviour exhibited by *trans*-**T** is typical^18^ for reactions mediated by autocatalytic processes, whereas, the spectroscopic behaviour exhibited by *trans*-**R** is more characteristic of a reaction that is either bimolecular or mediated^19^ by a binary reactive complex.

A concentration–time profile for the formation of both *trans*-**T** and *trans*-**R** (Fig. 8b) was constructed using deconvolution of these resonances, using tetrakis(trimethylsilyl) silane as an internal standard. The formation of the *cis* cycloadducts for the thread and rotaxane could not be monitored across the entire time course of the reaction as these resonances overlapped with others in the spectrum. Reaction of the equimolar solution of **L**, **S** and **M** (15 °C, CDCl_3_) resulted in 27% and 18% conversion to *trans*-**T** (maximum rate 0.47 mM h^–1^ at *t* = 1.25 h) and *trans*-**R** (maximum rate 0.49 mM h^–1^ at *t* = 0 h) cycloadducts after 15 hours, respectively. The ratio of *trans*-**T** (Fig. 8b, filled red circles) to *trans*-**R** (Fig. 8b, filled blue circles) was 1.5 : 1.

The sigmoidal rate profile (indicative of a self-replicating system) could not be unambiguously identified for either of the *trans* cycloadducts. In order to determine whether self-replication is operating within the rotaxane system, an experiment in which the reaction components were instructed with the preformed rotaxane *trans*-**R** was conducted. The addition of 38 mol% pre-synthesised rotaxane *trans*-**R** at the beginning of the reaction between **M**, **L** and **S** at 15 °C did not result in an increase in the conversion of either *trans*-**T** or *trans*-**R** (Fig 8c, empty red and blue diamonds, respectively) – reaching only 26% and 17% conversion, respectively, values mirroring closely those observed in the uninstructed kinetic experiment, giving a **T** : **R** ratio (1.5 : 1) that is identical to that determined in the absence of the added template. This result suggests that rotaxane *trans*-**R** is not capable of templating and accelerating its own formation *via* the catalytic quaternary [**L**·**M**·**S**·**R**] complex pathway, nor is it a suitable template for the formation of *trans*-**T**.

Additionally, we wanted to assess the role of recognition in the formation of the rotaxane through a reactive ternary complex [**L**·**M**·**S**]. To this end, we performed a kinetic experiment examining the reaction of **M**, **L** and **S** in the presence of 3.5 equivalents of 4-bromophenylacetic acid as an inhibitor (for details, see ESI†). The reaction resulted in a decrease in the conversion for both *trans*-**T** (18%) and *trans*-**R** (13%) after 15 hours, resulting in **T** : **R** of 1.4 : 1. The decrease in the conversion of both the thread and rotaxane in this control experiment clearly demonstrates that formation of both the thread and rotaxane is enhanced by the recognition processes within the system, and, in particular, suggests that the rotaxane could be formed *via* the [**L**·**M**·**S**] reactive complex.

**Fig. 9:**
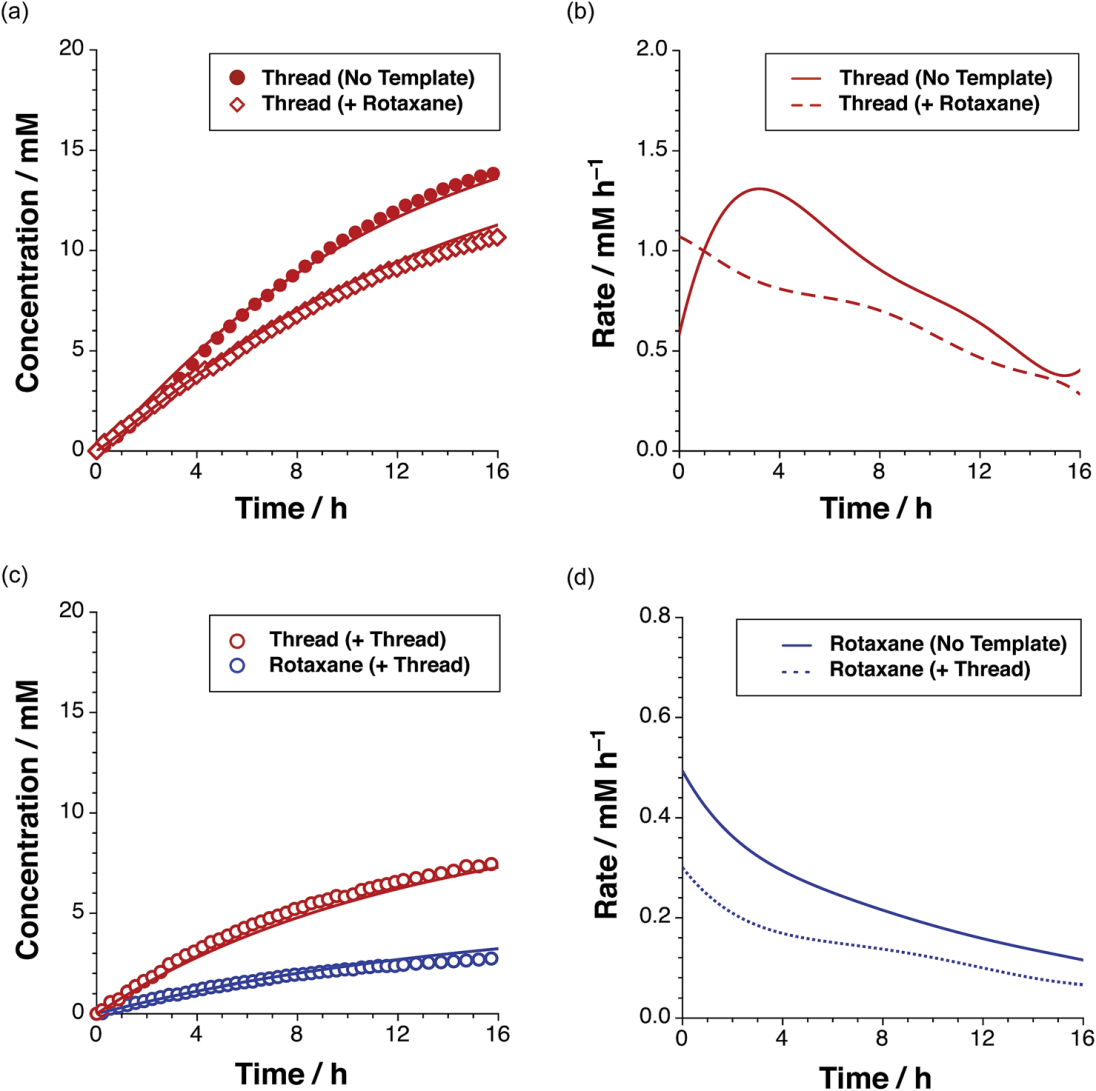
(a) Concentration *vs.* time and (b) rate *vs.* time profiles for the reaction of maleimide **L** with stopper **S** in the presence of 35 mol% preformed rotaxane *trans*-**R**, to give thread *trans*-**T** (empty red diamonds). (c) Concentration *vs.* time for the reaction of maleimide **L**, nitrone **S** and macrocycle **M** in the presence of 35 mol% pre-formed **T** (empty red/blue circles), as determined using 500 MHz ^1^H NMR spectroscopy (15 °C, CDCl_3_). (d) Rate *vs.* time profile for the formation of rotaxane in the presence of no instructed template (full blue line) and in the presence of 35 mol% *trans*-**T**.

In addition to the three thread (Fig. 3) and three rotaxane (Fig. 6) forming pathways described thus far, two crosscatalytic cycles potentially exist in this network – **R** can direct the formation of **T** and *vice versa*. In this experimental system, only *trans*-**T** was shown to be capable of catalysing its own formation *via* the template-mediated autocatalytic pathway. The results of the kinetic experiment examining rotaxane formation in the presence of the preformed rotaxane template (Fig. 8c) suggest that the rotaxane cannot cross-catalyse the formation of the thread. In order to confirm this result, we examined the reaction between maleimide **L** and nitrone **S** in the presence of 35 mol% pre-synthesised rotaxane **R** (Fig. 9a, empty red diamonds). The addition of **R** resulted in significantly lower conversion to *trans*-**T**, which reached only 52% after 15 h. This result suggests that the rotaxane template is acting as a non-specific inhibitor in the thread forming reaction. The recognition sites present in **R** act to sequester reagents into unproductive complexes in a manner similar to the competitive inhibitor experiments performed using 4-bromophenylacetic acid. The rate profile for the formation of the thread (Fig. 9b, dashed red line) changed noticeably, exhibiting a decrease in the maximum rate (1.07 mM h^–1^ at *t* = 0 h) relative to that observed in the uninstructed experiment (Fig. 9b, full red line). In order to probe the second cross-catalytic pathway, 35 mol% pre-synthesised thread *trans*-**T** was added to maleimide **L**, macrocycle **M** and nitrone **S** at the beginning of the reaction (Fig. 9c). Conversion to *trans*-**T** (Fig. 9c, empty red circles) increased to 37% after 15 h, yet again confirming the ability of the thread to template its own formation (maximum rate 0.58 mM h^–1^ at *t* = 0.55 h). The conversion to *trans*-**R** (Fig. 9c, empty blue circles), on the other hand, only reached 13% after 15 h and max. rate 0.30 mM h^–1^ at *t* = 0 h (Fig. 9d, blue dashed line).

The decrease in the formation of the rotaxane suggests that the thread template is not a suitable platform for the production of rotaxane in a recognition-mediated fashion. Taken together, these two experiments revealed that the rotaxane and thread are unable to catalyse the formation of each other.

In order to build a better understanding of the kinetic pathways active in the system and the parameters governing these pathways, we performed a kinetic simulation and fitting of all of the thread and rotaxane kinetic data acquired. Firstly, we constructed a model for the bimolecular formation of the thread in the absence of recognition elements (for details, see ESI†). Thus, we were able to extract the bimolecular rate constant (*k*_bi_) for the formation of *trans*-**T** and *cis*-**T**. Using the value of *k*_bi_ determined for the *trans* diastereoisomer, we next fitted the three recognition-enabled thread kinetic experiments simultaneously (–**T**, +**T**, +**R**, data in Fig. 4a and 9a), which allowed us to determine the rate constant^20^ for the template-directed unimolecular reaction (*k*_SR_), the thread duplex dimerisation constant^21^ (*K*_duplex_) and the corresponding effective molarity^22^ (Table 1).

**Table 1:** Kinetic parameters determined for the thread and rotaxane from fitting of the kinetic data shown in Fig. 4, 8 and 9 to the appropriate model. Simulation and fitting were performed using SimFit (G. von Kiedrowski, University of Bochum, 2008). The *K*_a_ for duplex [**T**·**T**] was determined to be 4.8 × 10^4^ M^–1^ and the *K*_a_ for the pseudorotaxane [**L**·**M**] was determined to be 220 M^–1^

	Thread	Rotaxane
	*trans*	*trans*
*k*_bi_/10^–4^ M^–1^ s^–1^	7.73	5.80
*k*_AB_/10^–4^ s^–1^	0.15	0.092
*k*_SR_/10^–4^ s^–1^	3.62	—
EM_AB_/mM	20	16
EM_SR_/mM	470	—

The rate constant for the cross-catalytic formation of the thread on the rotaxane template was not fitted, as we determined through our kinetic analyses that this pathway does not operate in this system. The fitting procedure was repeated^23^ for the rotaxane kinetic data. In this instance however, only *k*_AB_ for the recognition-mediated formation of the rotaxane through the reactive complex [**L**·**M**·**S**] was fitted, as the kinetic analyses revealed that the rotaxane is not capable of establishing either an auto- or cross-catalytic reaction pathway. An overview of the kinetic parameters determined through this fitting procedure is presented in Table 1 and the best fits of the appropriate models to the experimental data are shown as solid lines in Fig. 4, 8 and 9. In the case of the thread, *trans*-**T**, it is clear that the major pathway exploited in the formation of this product is the autocatalytic template-mediated pathway. The value of EM_SR_ – 470 mM – is considerably lower than that observed7d,^11^,^12^,^15^ (2 → 200 M) in other replicating systems studied in our laboratory. This observation probably reflects the increased flexibility of *trans*-**T** when compared to the other autocatalytic templates we have reported. The fitting of the kinetic data also reveals that the pathway involving the binary reactive complex [**L**·**S**] (EM_AB_ = 20 mM) has a minimal role in the formation of *trans*-**T**.

**Fig. 10:**
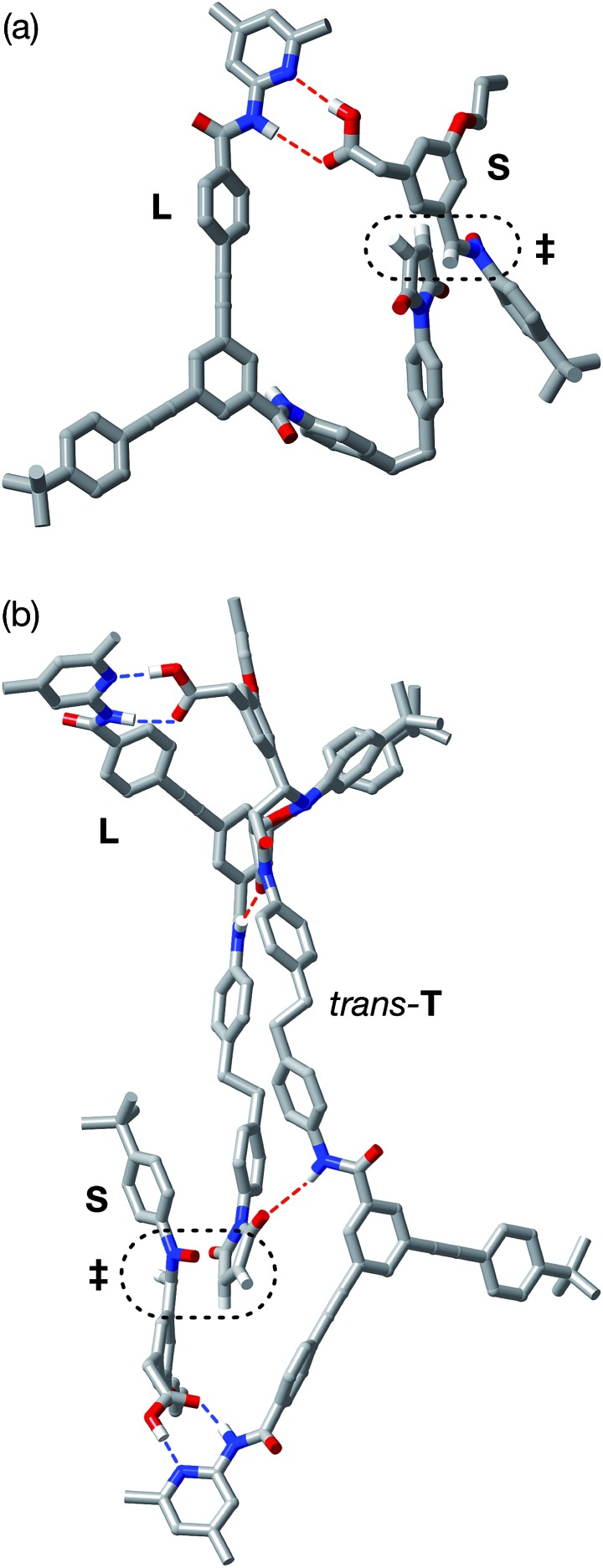
(a) Stick representation of the computed transition state geometry (RM1) leading to the formation of *trans*-**T′** formed through the [**L**·**S**] complex. (b) Stick representation of the computed transition state geometry (RM1) leading to the formation of [*trans*-**T**·*trans*-**T**] through the ternary complex [**L**·**S**·**T**]. Carbon atoms are coloured grey, nitrogen atoms are coloured blue, oxygen atoms are coloured red and hydrogen atoms are coloured white. For clarity, most hydrogen atoms are not shown. Hydrogen bonds with D–H···A distances <2.0 Å are shown as blue dashed lines, hydrogen bonds with D–H···A distances between 2.0 and 2.5 Å are shown as red dashed lines. The transition states for the forming isoxazolidine rings are enclosed in the black dotted lines.

In order to assess the plausibility of these two recognition-mediated pathways for the formation of *trans*-**T**, we performed a series of calculations. We located the transition states for the formation of *trans*-**T** from the binary complex [**L**·**S**] and the ternary complex [**L**·**S**·**T**] using the RM1 (ref. 24) method. Although the binary complex [**L**·**S**] can access (Fig. 10a) a plausible transition state (*r*_C–O_ = 2.03 Å; *r*_C–C_ = 2.13 Å; *ν*_i_ = 543 cm^–1^), this structure exhibits significant extensions (*r*_N···HO_ = 2.40 Å; *r*_NH···O_ = 2.48 Å) of both the hydrogen bonding distances in the key amidopyridine·carboxylic acid recognition element away from their optimum values. This disruption of the hydrogen bonds limits the efficiency of the [**L**·**S**] complex as a vehicle for the formation of *trans*-**T**. By contrast, the ternary complex [**L**·**S**·**T**] can access (Fig. 10b) a transition state in which, in addition to interactions formed between the amidopyridines and carboxylic acids (average *r*_N···HO_ = 1.57 Å; *r*_NH···O_ = 1.71 Å), there are a number of additional stabilising and polarising^25^ interactions between the amide NH protons and the carbonyl oxygen atoms of the maleimide and isoxazolidine rings. The structure of the transition state associated with the forming isoxazolidine ring is as expected (*r*_C–O_ = 2.03 Å; *r*_C–C_ = 2.14 Å; *ν*_i_ = 571 cm^–1^).

By contrast, rotaxane *trans*-**R** barely benefits from recognition-mediated assistance at all in its formation – the value of EM_AB_ is the same as the reaction concentration. It is likely that the bimolecular pathway and the pathway mediated by the [**L**·**M**·**S**] complex are equally important in the formation of *trans*-**R**. This hypothesis is supported by the fact that, although *trans*-**R** is not formed at a rate significantly higher than the bimolecular cycloaddition, the diastereoselectivity of the reaction forming *trans*-**R** is significantly better. However, attempts to fit the observed kinetic profiles involving rotaxane formation using a model that excludes the ternary complex pathway afforded catastrophically poor fits.

**Fig. 11:**
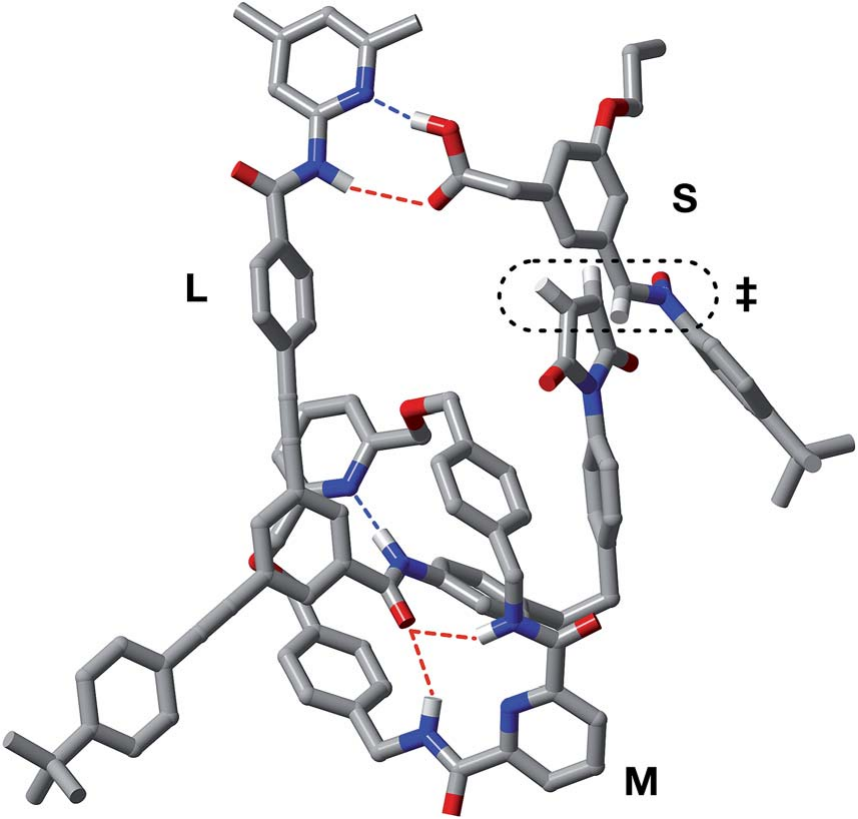
Stick representation of the computed transition state geometry (RM1) for the formation of *trans*-**R** through the [**L**·**M**·**S**] complex. Carbon atoms are coloured grey, nitrogen atoms are coloured blue, oxygen atoms are coloured red and hydrogen atoms are coloured white. For clarity, most hydrogen atoms are not shown. Hydrogen bonds with D–H···A distances <2.0 Å are shown as blue dashed lines, hydrogen bonds with D–H···A distances between 2.0 and 2.5 Å are shown as red dashed lines. The transition state for the forming isoxazolidine ring is enclosed in the black dotted line.

In order to assess potential issues with the two recognition-mediated pathways for the formation of *trans*-**R**, we, once again, performed a series of calculations. We located the transition state for the formation of *trans*-**R** from the ternary complex [**L**·**M**·**S**] using the RM1 (ref. 24) method. Although the ternary complex [**L**·**M**·**S**] can access (Fig. 11) a plausible transition state (*r*_C–O_ = 2.02 Å; *r*_C–C_ = 2.14 Å; *ν*_i_ = 556 cm^–1^), this structure, in common with the structure discussed above in the context of the [**L**·**S**] complex, also has a significant distortion of the key amidopyridine·carboxylic acid recognition motif. However, in this case, the hydrogen bond between the pyridine ring and the acid proton is intact (*r*_N···HO_ = 1.61 Å), but the hydrogen bond between the amide proton and the carbonyl oxygen is lengthened significantly (*r*_NH···O_ = 2.37 Å). This distortion of the critical recognition element probably limits the efficiency of this complex as a vehicle for the formation of *trans*-**R**. It proved impossible to locate a plausible transition state structure for the formation of *trans*-**R** from the quaternary complex [**L**·**M**·**S**·**R**]. The location of a macrocycle at each of the two amide binding sites cannot be accommodated within a structure similar to that calculated for [**L**·**M**·**S**·**R**]. The presence of the macrocycle also prevents the formation of the additional stabilising and polarising hydrogen bonds between the amide NH protons and the carbonyl oxygen atoms of the maleimide and isoxazolidine rings with [**L**·**M**·**S**·**R**] that appear to be critical to the successful operation of the [**L**·**S**·**T**] ternary complex. This observation may also help to explain the absence of any crosscatalysis between *trans*-**T** and *trans*-**R**.

## Conclusions

In conclusion, we have described the full characterisation of a reaction network that integrates recognition-mediated reaction processes with the assembly of a [2]rotaxane. The initial goals of this research were to address two critical shortcomings of our previous, successful design of a replicating [2]rotaxane. The issues associated with the unproductive binding modes have been addressed completely in the new design reported here. In order to remove unwanted crosscatalytic relationships between the [2]rotaxane and the corresponding thread, the macrocyclic component was relocated into a central location in the design of the [2]rotaxane (Fig. 2). We envisaged that, by placing the macrocyclic component **M** in this location, discrimination between the catalytic cycles that constructed the [2]rotaxane, *trans*-**R**, and those associated with the corresponding thread, *trans*-**T**, would be enhanced. However, the outcome of this design change was only partly successful. While thread **T** can template its own formation and does not participate in any crosscatalytic pathways, the formation of the rotaxane did not proceed through an autocatalytic cycle, but rather through a relatively inefficient ternary complex reaction channel. Kinetic fitting and semi-empirical electronic structure calculations, traced these issues to the unwanted distortion of one of the key recognition elements in the system. Additionally, the presence of a flexible spacer at the heart of the molecular design compounded these issues by allowing the unwanted folding of the rotaxane and thread structures, thereby hampering their ability to act as templates. This structural flexibility can be removed by a careful structural redesign of the components of this system, in particular maleimide **L**, and this work is currently underway in our laboratory.

## Supplementary Material

Supplementary information
