# Supplementary material for: Exploiting recognition-mediated assembly and reactivity in [2]rotaxane formation
Source: Chem Sci. 2016 Jan 15;7(4):2592–603. doi: 10.1039/c5sc04805b (PMC5477148; doi:10.1039/c5sc04805b)
Supplement: Supplementary file 1 [file SC-007-C5SC04805B-s001.pdf]

Electronic Supplementary Material (ESI) for Chemical Science.
This journal is © The Royal Society of Chemistry 2016

Chemical Science

Exploiting recognition-mediated assembly and reactivity in [2]rotaxane formation

Annick Vidonne[a], Tamara Kosikova[a] and Douglas Philp[*a]

School of Chemistry and EaStCHEM, University of St Andrews, North Haugh, St Andrews, Fife KY16 9ST, United Kingdom

Corresponding author: d.philp@st-andrews.ac.uk

Supplementary Information

Table of Contents

1. General procedures **2**

2. Synthetic procedures **4**
*2.1 Synthesis of macrocycle **M*** *4*
*2.2 Synthesis of maleimide **L*** *7*
*2.3 Synthesis of rotaxane **R** and thread **T*** *9*
2.4 Synthesis of recognition-disabled nitrone $\mathbf{S}_{dis}$ *11*

3. Kinetic studies **13**
*3.1 ^{1}H NMR spectroscopic binding study between **M** and **L*** *13*
3.2 Thread recognition-disabled kinetic experiments *14*
3.3 Rotaxane recognition-disabled kinetic experiment *15*

4. Example SimFit fitting scripts **16**
*4.1 SimFit input file for the bimolecular reaction of **L** with recognition-disabled nitrone* $\mathbf{S}_{dis}$ *16*
*4.2 SimFit input file for thread forming reaction of **L** with **S*** *17*
*4.3 SimFit input file for thread and rotaxane forming reaction of **L** with **S** in the presence of **M*** *19*

5. References **21**

6. XYZ Files for computed transition state geometries **22**
*6.1 Computed transition state geometry for trans-**T** formed through binary complex* *22*
*6.2 Computed transition state geometry for trans-**T** formed through ternary complex* *25*
*6.3 Computed transition state geometry for trans-**R** formed through ternary complex* *30*

7. NMR spectroscopic data **33**

1. General procedures

Chemicals and solvents were purchased from standard commercial suppliers and were used as received unless otherwise stated. Tetrahydrofuran was dried by heating to reflux in the presence of sodium/benzophenone under a nitrogen atmosphere and was collected by distillation. Dichloromethane was dried by heating under reflux over calcium hydride and distilled under nitrogen.

Reactions were monitored by thin-layer chromatography carried out on 0.2 mm MP Biomedicals pre-coated aluminium backed silica sheets using UV light as visualizing agent, and ninhydrin, 2,4-dinitrophenylhydrazine and potassium permanganate solution as developing agents. Column chromatography was performed using Kieselgel 60 (0.040–0.063 mm mesh, Merck 9385) or MP Silica (silica gel, 0.032–0.063 mesh).

Chemical ionization mass spectrometry (CI) at low and high resolution was carried out using a Micromass GCT spectrometer. Electrospray mass spectrometry (ES) at low and high resolution was carried out using a Micromass LCT spectrometer.

NMR spectra were recorded on either a Bruker Avance 300, or a Bruker Avance II 400, or a Bruker Avance 500, or a Varian UnityPLUS 500 spectrometer using deuterated solvent as the lock and the residual solvent as the internal reference in all cases. All spectra were recorded at 298 K unless otherwise stated. Chemical shits δ are reported in parts per million (ppm). All coupling constants J are quoted to the nearest 0.1 Hz. In the assignment of ^{1}H NMR spectra the symbols br, s, d, t, q and m denote broad, singlet, doublet, triplet, quartet and multiplet, respectively. In the assignment of ^{13}C NMR spectra the symbol *quat*. denotes a quaternary carbon. ^{1}H and ^{13}C data were assigned by a combination of one- and two-dimensional experiments (COSY, HSQC, HMBC, HMQC, ROESY).

For the kinetic measurements, masses of reagents were measured using a Sartorius BP 211D balance (± 0.01 mg) and reagent solutions prepared by dissolving compounds in the appropriate volume of $CDCl_3$. Subsequent experimental samples, suitable for kinetic experiments, were prepared by using Hamilton gas-tight syringes to transfer solution to a Wilmad 528PP NMR tube, which was then fitted with a polyethylene pressure cap to minimise solvent evaporation. When required, concentrations were calibrated using $(Me_3Si)_4Si$ as internal standard, added to the reaction mixtures at a concentration of 0.5 mM. Reaction mixtures were monitored systematically by 500 MHz ^{1}H NMR spectroscopy over 16 or 24 hours. ^{1}H NMR spectra were acquired automatically every 20 or 30 minutes. Analysis and deconvolution of the appropriate resonances of each of the ^{1}H NMR spectra was performed using Bruker Topspin software (version 2.0, 2006 or iNMR 5.4.7, Mestrelab Research).

Electronic structure calculations were carried out using GAMESS-US[1,2] and the 64-bit Linux version dated 5 Dec 2014 (Revision 1) compiled from source (gfortran 4.4.7; ATLAS 3.8.4) on CentOS 6.7 was used in all calculations. The following workflow was used to locate each of the transition state structures reported.

1. An initial geometry was constructed and optimised using RM1[3] with the lengths of the forming C–O and C–C bonds in the isoxazolidine ring fixed.
2. The Hessian matrix for this structure was calculated numerically at the RM1 level of theory using the keyword `RUNTYP=HESSIAN`.
3. This Hessian matrix was used to direct the location of the transition state at the RM1 level of theory using the keyword `RUNTYP=SADPOINT`.
4. The presence of one imaginary vibrational frequency corresponding to the reaction coordinate in the optimised transition state structure was verified by vibrational analysis.

2. Synthetic procedures

2.1 Synthesis of macrocycle M

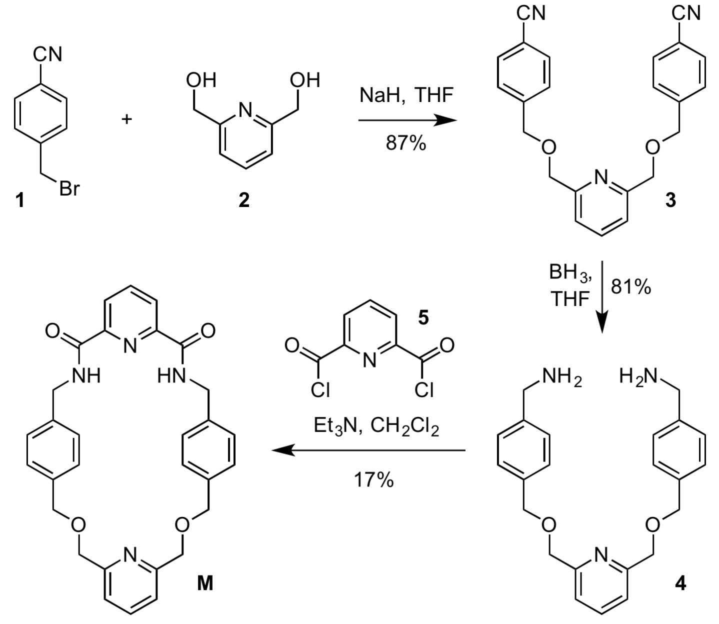

4,4'-[Pyridine-2,6-diylbis(methyleneoxymethylene)]dibenzonitrile 3[4]

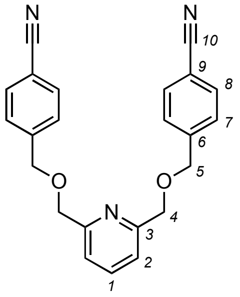

NaH (60% w/w dispersion in mineral oil, 1.87 g, 46.8 mmol) was slowly added to a mixture of 4-(bromomethyl)benzonitrile **1** (7.40 g, 37.7 mmol) and 2,6-pyridinedimethanol **2** (2.50 g, 18.0 mmol) in dry THF (120 mL). The reaction mixture was heated at 70 ºC for 2 days, and then quenched with water. The mixture was concentrated by evaporation under reduced pressure and extracted with EtOAc (3 ×). The combined organic layers were dried ($MgSO_4$) and the solvent was removed by evaporation under reduced pressure. The residue was purified by column chromatography (SiO_2, cyclohexane/EtOAc 1:1) to give **3** as a white solid (5.77 g, 87%). ^{1}H NMR (400.1 MHz, $CDCl_3$): δ = 7.75 (t, J = 7.7 Hz, 1H, H_1), 7.67–7.64 (m, AA' of AA'BB' system, 4H, H_8), 7.51–7.48 (m, BB' of AA'BB' system, 4H, H_7), 7.40 (d, J = 7.7 Hz, 2H, H_2), 4.71 (s, 4H, H_5), 4.70 (s, 4H, H_4); ^{13}C NMR (100.6 MHz, $CDCl_3$): δ = 157.6 (quat. C, C_3), 143.6 (quat. C, C_6), 137.6 (CH, C_1), 132.4 (CH, C_8), 127.9 (CH, C_7), 120.4 (CH, C_2), 118.9 (quat. C, C_{10}), 111.6 (quat. C, C_9), 73.7 (CH_2, C_4), 72.1 (CH_2, C_5); MS (CI+): *m/z* (%) = 239 (27), 370 ($[M+H]^+$, 100); HRMS (CI+): *m/z* calc. for $[M+H]^+$ $C_{23}H_{20}N_3O_2$ 370.1556, found 370.1544.

(4,4'-(Pyridine-2,6-diylbis(methylene))bis(oxy)bis(methylene)bis(4,1-phenylene))dimethanamine 4[4]

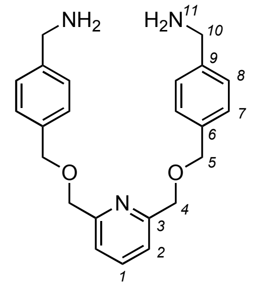

A solution of BH_3•THF complex (1 M solution in THF, 29 mL, 29.0 mmol) was added to a solution of **3** (2.35 g, 6.4 mmol) in dry THF (80 mL) at 0 °C under a N_2 atmosphere. The reaction mixture was stirred at ambient temperature for 1.5 h, and then heated at 70 ºC for 18 h. The reaction mixture was quenched with 1 M HCl, concentrated by evaporation under reduced pressure and extracted with CH_2Cl_2 (1 ×). The aqueous layer was made alkaline with 1 M KOH (up to pH 12) and extracted with CH_2Cl_2 (3 ×). The combined organic layers were dried ($MgSO_4$) and the solvent was removed by evaporation under reduced pressure to yield the desired **4** (1.96 g, 81%) as a white solid, which was used in the next step without further purification. ^1H NMR (400.1 MHz, $CDCl_3$): δ = 7.71 (t, J = 7.7 Hz, 1H, H_1), 7.39 (d, J = 7.8 Hz, 2H, H_2), 7.36 (d, AA' of AA'BB' system, J = 8.2 Hz, 4H, H_7), 7.30 (d, BB' of AA'BB' system, J = 8.2 Hz, 4H, H_8), 4.66 (s, 4H, H_4), 4.63 (s, 4H, H_5), 3.87 (s, 4H, H_{10}), 1.46 (br s, 4H, H_{11}); ^{13}C NMR (75.5 MHz, $CDCl_3$): δ = 158.1 (quat. C, C_3), 143.1 (quat. C, C_9), 137.4 (CH, C_1), 136.6 (quat. C, C_6), 128.3 (CH, C_7), 127.3 (CH, C_8), 120.1 (CH, C_2), 73.2 (CH_2, C_4), 72.9 (CH_2, C_5), 46.5 (CH_2, C_{10}); MS (ES+): m/z (%) = 374 (50), 378 ($[M+H]^+$, 100), 400 (47); HRMS (ES+): m/z calc. for $[M+Na]^+$ $C_{23}H_{27}N_3O_2Na$ 400.2001, found 400.2005.

Macrocycle M[4]

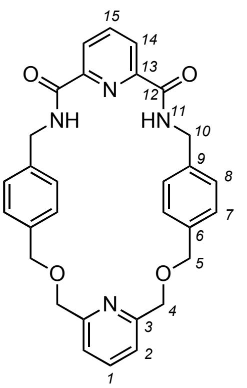

The crude diamine **4** (1.00 g) was dissolved in dry CH_2Cl_2 (110 mL) and was added simultaneously with a solution of 2,6-pyridinedicarbonyl dichloride **5** (0.54 g, 2.6 mmol) in dry CH_2Cl_2 (110 mL) to a

solution of Et_3N (1.5 mL, 10.8 mmol) in dry CH_2Cl_2 (110 mL) over 1.5 h at ambient temperature under a N_2 atmosphere. The reaction mixture was stirred at ambient temperature for 4 days, and then heated to reflux for 3 more days. The reaction mixture was washed successively with 1 M HCl and 1 M KOH. The organic layer was dried ($MgSO_4$) and the solvent was removed by evaporation under reduced pressure. The residue was purified by column chromatography (SiO_2, gradient from 45:45:10 to 0:90:10 cyclohexane/EtOAc/$CHCl_3$) to give **M** as a white solid (227 mg, 17%). ^{1}H NMR (400.1 MHz, $CDCl_3$): δ = 8.40 (d, J = 7.8 Hz, 2H, H_{14}), 8.06 (dd, J = 8.0, 7.6 Hz, 1H, H_{15}), 7.88 (br t, J = 5.8 Hz, 2H, H_{11}), 7.70 (t, J = 7.7 Hz, 1H, H_1), 7.34–7.31 (m, 6H, H_2+H_7), 7.28–7.26 (m, 4H, H_8), 4.70 (d, J = 6.1 Hz, 4H, H_{10}), 4.62 (s, 4H, H_5), 4.52 (s, 4H, H_4); ^{13}C NMR (100.6 MHz, $CDCl_3$): δ = 163.4 (quat. C, C_{12}), 157.6 (quat. C, C_3), 148.9 (quat. C, C_{13}), 139.3 (CH, C_{15}), 137.5 (quat. C, C_9), 137.4 (quat. C, C_6), 137.3 (CH, C_1), 129.1 (CH, C_7), 127.9 (CH, C_8), 125.5 (CH, C_{14}), 121.2 (CH, C_2), 72.0 (CH_2, C_4), 71.8 (CH_2, C_5), 43.3 (CH_2, C_{10}); MS (ES+): m/z (%) = 531 ([M+Na]$^+$, 100), 532 (60); HRMS (ES+): m/z calc. for [M+Na]$^+$ $C_{30}H_{28}N_4O_4Na$ 531.2008, found 531.2009.

2.2 Synthesis of maleimide L

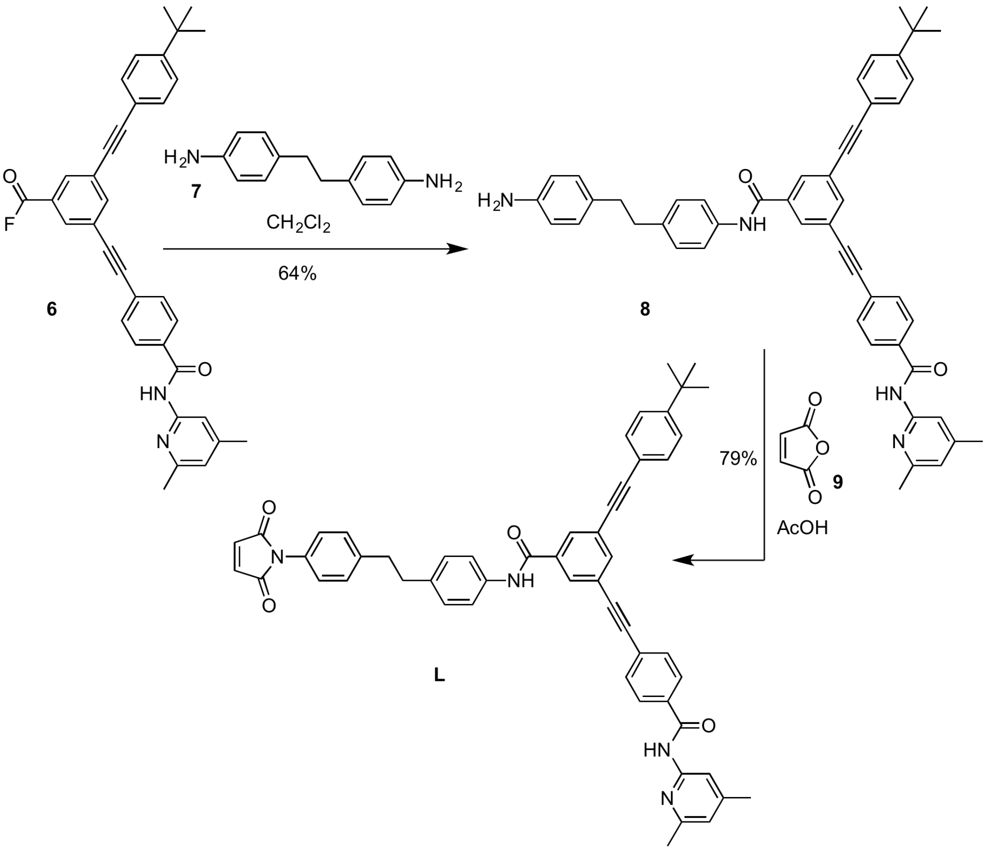

Maleimide L

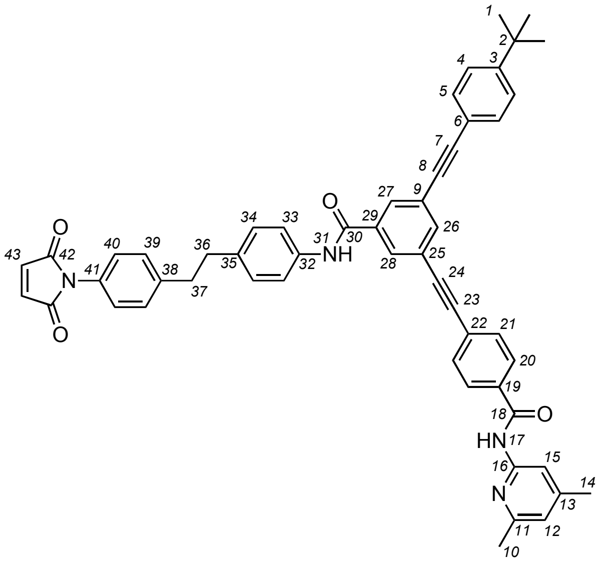

A solution of acid fluoride **6**[5] (0.909 g, 1.72 mmol) in dry CH_2Cl_2 (20 mL) was added dropwise over 50 min under a N_2 atmosphere to a solution of 4,4'-ethylenedianiline **7** (1.108 g, 5.22 mmol) in dry CH_2Cl_2 (20 mL). The reaction mixture was stirred at ambient temperature overnight, and then diluted with CH_2Cl_2. The mixture was washed successively with saturated $NaHCO_3$ solution and saturated NaCl solution, dried ($MgSO_4$) and concentrated by evaporation under reduced pressure. The residue was purified by column chromatography (SiO_2, gradient from 100:0 to 99:1 CH_2Cl_2/MeOH) to give the

amine **8** (64% NMR yield). The product, contaminated with traces of 4,4'-ethylenedianiline, was used in the next step without further purification. Maleic anhydride **9** (98 mg, 0.999 mmol) was added to a solution of **8** (0.832 mmol) in acetic acid (10 mL). The reaction mixture was stirred at ambient temperature for 6 h, and then heated at 125 ºC for 16 h. The solvent was removed by evaporation under reduced pressure. The residue was purified by column chromatography (SiO_2, gradient from 100:0 to 99.5:0.5 $CHCl_3$/MeOH) to give **L** (528 mg, 79%) as a yellow solid. ^1H NMR (400.1 MHz, $CDCl_3$): δ = 8.46 (s, 1H, H_{17}), 8.03 (s, 1H, H_{15}), 7.97 (d, J = 1.5 Hz, 2H, H_{27}+H_{28}), 7.95–7.92 (m, AA' of AA'BB' system, 2H, H_{20}), 7.87 (s, 1H, H_{31}), 7.85 (t, J = 1.5 Hz, 1H, H_{26}), 7.66–7.64 (m, BB' of AA'BB' system, 2H, H_{21}), 7.60–7.57 (m, AA' of AA'BB' system, 2H, H_{33}), 7.51–7.47 (m, AA' of AA'BB' system, 2H, H_5), 7.42–7.39 (m, BB' of AA'BB' system, 2H, H_4), 7.29–7.24 (m, AA'BB' system, 4H, H_{39}+H_{40}), 7.22–7.19 (m, BB' of AA'BB' system, 2H, H_{34}), 6.85 (s, 2H, H_{43}), 6.79 (s, 1H, H_{12}), 2.97–2.93 (m, 4H, H_{36}+H_{37}), 2.44 (s, 3H, H_{10}), 2.36 (s, 3H, H_{14}), 1.34 (s, 9H, H_1); ^{13}C NMR (75.5 MHz, $CDCl_3$): δ = 169.8 (quat. C, C_{42}), 164.9 (quat. C, C_{18}), 164.4 (quat. C, C_{30}), 156.6 (quat. C, C_{11}), 152.3 (quat. C, C_3), 150.7 (quat. C, C_{16}), 150.3 (quat. C, C_{13}), 141.7 (quat. C, C_{38}), 138.0 (quat. C, C_{35}), 137.1 (CH, C_{26}), 136.0 (quat. C), 135.9 (quat. C, C_{32}), 134.3 (CH, C_{43}), 134.1 (quat. C, C_{19}), 132.1 (CH, C_{21}), 131.6 (CH, C_5), 130.2 (CH, C_{27} or C_{28}), 129.7 (CH, C_{27} or C_{28}), 129.3 (CH, C_{39}), 129.2 (CH, C_{34}), 129.1 (quat. C, C_{41}), 127.4 (CH, C_{20}), 126.6 (quat. C, C_{22}), 126.1 (CH, C_{40}), 125.6 (CH, C_4), 124.8 (quat. C), 123.8 (quat. C), 121.0 (CH, C_{12}), 120.6 (CH, C_{33}), 119.6 (quat. C, C_6), 111.8 (CH, C_{15}), 91.7 (quat. C, C_7), 90.6 (quat. C, C_8 or C_{24}), 90.1 (quat. C, C_{23}), 87.1 (quat. C, C_8 or C_{24}), 37.6 (CH_2, C_{37}), 37.3 (CH_2, C_{36}), 35.0 (quat. C, C_2), 31.3 (CH_3, C_1), 23.9 (CH_3, C_{10}), 21.4 (CH_3, C_{14}); MS (ES+): m/z (%) = 823 ($[M+Na]^+$, 100); HRMS (ES+): m/z calc. for $[M+H]^+$ $C_{53}H_{45}N_4O_4$ 801.3441, found 801.3429.

2.3 Synthesis of rotaxane R and thread T

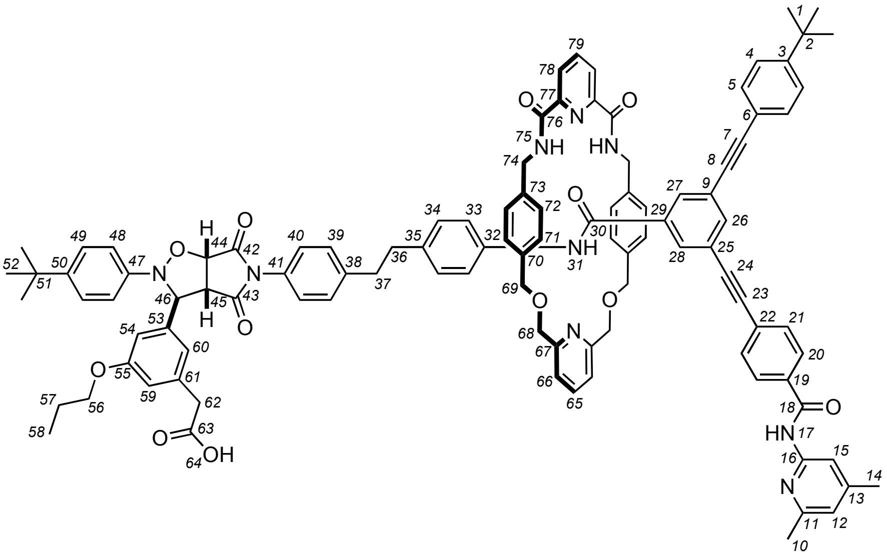

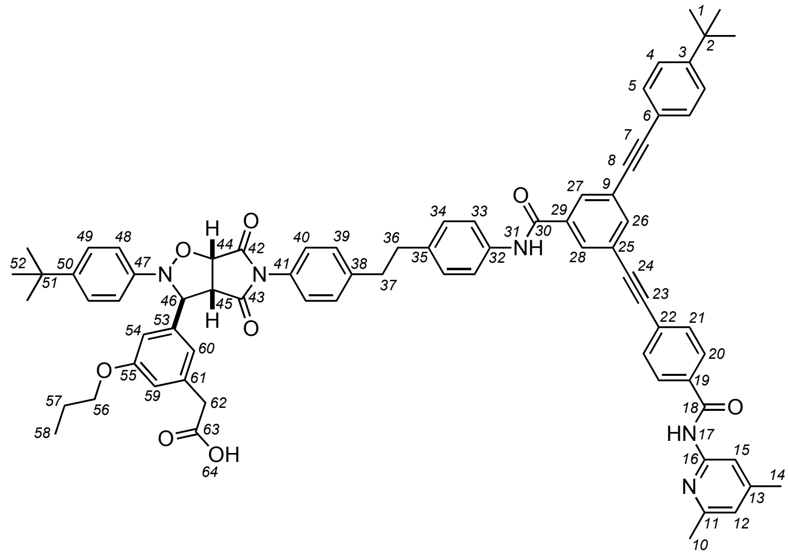

A mixture of macrocycle **M** (208 mg, 0.41 mmol) and maleimide **L** (329 mg, 0.41 mmol) in $CHCl_3$ (9.0 mL) was stirred at ambient temperature for 1 h under a N_2 atmosphere. Nitrone **S**[1] (151 mg, 0.41 mmol) was then added to the reaction mixture. The reaction mixture was stirred at ambient temperature under a N_2 atmosphere in the absence of light for 6 days. The solvent was removed by evaporation under reduced pressure. The residue was purified by careful column chromatography (SiO_2, gradient cyclohexane / EtOAc), which enabled the isolation of the different products in the following proportions: rotaxane **R** (317 mg, 46%, *trans*/*cis* 18:1) as a white solid and thread **T** (218 mg, 45%, *trans*/*cis* 35:1) as a white solid.

Representative data for rotaxane (*trans*-**R**): ^{1}H NMR (499.9 MHz, $CDCl_3$): δ = 10.39 (br s, 1H, H_{17}), 9.46 (t, J = 5.9 Hz, 1H, H_{75}), 9.42 (t, J = 5.7 Hz, 1H, $H_{75'}$), 9.18 (s, 1H, H_{31}), 8.48 (dd, J = 7.8, 1.0 Hz, 1H, H_{78}), 8.44 (dd, J = 7.9, 1.1 Hz, 1H, $H_{78'}$), 8.16 (s, 1H, H_{15}), 8.02 (d, AA' of AA'BB' system, J = 8.4 Hz, 2H, H_{20}), 7.94 (t, J = 7.8 Hz, 1H, H_{79}), 7.71 (t, J = 7.7 Hz, 1H, H_{65}), 7.61 (s, 1H, H_{27} or H_{28}), 7.55 (s, 1H, H_{26}), 7.49 (s, 1H, H_{27} or H_{28}), 7.40 (d, BB' of AA'BB' system, J = 8.4 Hz, 2H, H_{21}), 7.40 (d, AA' of AA'BB' system, J = 8.6 Hz, 2H, H_4), 7.35 (d, BB' of AA'BB' system, J = 8.5 Hz, 2H, H_5), 7.32 (d, J = 8.0 Hz, 2H, H_{66}), 7.30 (d, AA' of AA'BB' system, J = 8.6 Hz, 2H, H_{33}), 7.18 (d, AA' of AA'BB' system, J = 8.9 Hz, 2H, H_{49}), 7.14 (s, 1H, H_{54}), 7.08 (d, BB' of AA'BB' system, J = 8.9 Hz, 2H, H_{48}), 7.07 (s, 1H, H_{60}), 6.98 (s, 1H, H_{59}), 6.95 (d, AA' of AA'BB' system, J = 8.3 Hz, 2H, H_{39}), 6.82 (s, 1H, H_{12}), 6.80–6.73 (m, 8H, H_{72}+H_{71}), 6.61 (d, BB' of AA'BB' system, J = 8.5 Hz, 2H, H_{34}), 6.36 (d, BB' of AA'BB' system, J = 8.3 Hz, 2H, H_{40}), 5.74 (s, 1H, H_{46}), 5.07 (d, J = 7.6 Hz, 1H, H_{44}), 4.64–4.48 (m, 4H,

H_{74}), 4.46–4.34 (m, 4H, H_{69}), 4.34–4.16 (m, 4H, H_{68}), 3.98 (d, J = 7.6 Hz, 1H, H_{45}), 3.96–3.93 (m, 2H, H_{56}), 3.68 (s, 2H, H_{62}), 2.89–2.78 (m, 4H, H_{37}+H_{36}), 2.49 (s, 3H, H_{10}), 2.40 (s, 3H, H_{14}), 1.84–1.77 (m, 2H, H_{57}), 1.36 (s, 9H, H_{1}), 1.22 (s, 9H, H_{52}), 1.03 (t, J = 7.4 Hz, 3H, H_{58}); ^{13}C NMR (100.6 MHz, $CDCl_3$): δ = 176.1 (quat. C, C_{63}), 174.2 (quat. C, C_{43}), 172.9 (quat. C, C_{42}), 166.0 (quat. C, C_{18}), 164.2 (quat. C, C_{76}), 164.2 (quat. C, $C_{76'}$), 164.0 (quat. C, C_{30}), 160.1 (quat. C, C_{55}), 157.3 (quat. C, C_{67}), 157.2 (quat. C, $C_{67'}$), 155.5 (quat. C, C_{11}), 152.2 (quat. C, C_{3}), 152.1 (quat. C, C_{13}), 151.2 (quat. C, C_{16}), 149.3 (quat. C, C_{77}), 146.8 (quat. C, C_{47}), 145.9 (quat. C, C_{50}), 142.5 (quat. C, C_{38}), 141.3 (quat. C, C_{53}), 138.8 (CH, C_{79}), 137.7 (quat. C, C_{73}), 137.7 (CH, C_{65}), 137.5 (quat. C, $C_{73'}$), 136.8 (quat. C, C_{32}), 136.6 (quat. C, C_{61}), 136.0 (quat. C, C_{35}), 135.7 (CH, C_{26}), 135.6 (quat. C, C_{70}), 135.5 (quat. C, $C_{70'}$), 134.6 (quat. C), 133.8 (quat. C, C_{19}), 131.9 (CH, C_{21}), 131.6 (CH, C_{27} or C_{28}), 131.6 (CH, C_{5}), 131.6 (CH, C_{27} or C_{28}), 129.5 (CH, C_{39}), 129.1 (CH, C_{71}), 129.0 (CH, $C_{71'}$), 128.9 (quat. C, C_{41}), 128.4 (CH, C_{34}), 128.3 (CH, C_{72}), 128.1 (2 × CH, C_{20}+$C_{72'}$), 126.7 (quat. C, C_{22}), 126.3 (CH, C_{49}), 126.1 (CH, C_{40}), 125.5 (CH, C_{4}), 125.4 (CH, C_{78}), 123.3 (quat. C), 122.1 (quat. C), 121.7 (CH, C_{66}), 121.3 (CH, C_{12}), 119.8 (2 × CH, C_{33}+C_{60}), 119.7 (quat. C, C_{6}), 115.0 (CH, C_{59}), 114.1 (CH, C_{48}), 113.5 (CH, C_{15}), 111.1 (CH, C_{54}), 90.6 (quat. C, C_{7}), 90.1 (quat. C, C_{8} or C_{24}), 89.2 (quat. C, C_{23}), 87.0 (quat. C, C_{8} or C_{24}), 77.8 (CH, C_{44}), 73.0 (CH_2, C_{69}), 72.8 (CH_2, $C_{69'}$), 71.3 (CH_2, C_{68}), 71.0 (CH_2, $C_{68'}$), 70.1 (CH, C_{46}), 69.8 (CH_2, C_{56}), 57.8 (CH, C_{45}), 43.7 (CH_2, C_{74}), 43.5 (CH_2, $C_{74'}$), 42.5 (CH_2, C_{62}), 37.6 (CH_2, C_{37}), 37.0 (CH_2, C_{36}), 35.0 (quat. C, C_{2}), 34.2 (quat. C, C_{51}), 31.5 (CH_3, C_{52}), 31.3 (CH_3, C_{1}), 22.7 (CH_2, C_{57}), 22.4 (CH_3, C_{10}), 21.7 (CH_3, C_{14}), 10.7 (CH_3, C_{58}); HRMS (MALDI): *m/z* calc. for $[M+H]^+$ $C_{105}H_{100}N_9O_{12}$ 1678.7491, found 1678.7057.

Representative data for thread (*trans*-**T**): ^{1}H NMR (400.1 MHz, $CDCl_3$): δ = 10.96 (br s, 1H, NH), 8.80 (s, 1H, NH), 8.34 (s, 1H, H_{27} or H_{28}), 8.28 (s, 1H, H_{27} or H_{28}), 8.19 (s, 1H, H_{15}), 7.88–7.86 (m, 3H, H_{26}+H_{20}), 7.51–7.48 (m, 4H, H_{5}+H_{33}), 7.42–7.39 (m, BB' of AA'BB' system, 2H, H_{4}), 7.30 (d, BB' of AA'BB' system, J = 8.4 Hz, 2H, H_{21}), 7.22 (s, 1H, H_{54}), 7.08–6.97 (m, 7H, H_{60}+H_{49}+H_{39}+H_{48}), 6.91–6.89 (m, 3H, H_{34}+H_{59}), 6.82 (s, 1H, H_{12}), 6.25 (d, BB' of AA'BB' system, J = 8.4 Hz, 2H, H_{40}), 5.85 (s, 1H, H_{46}), 5.29 (d, J = 7.6 Hz, 1H, H_{44}), 4.03–3.92 (m, 3H, H_{45}+H_{56}), 3.71 (s, 2H, H_{62}), 2.90–2.80 (m, 4H, H_{36}+H_{37}), 2.50 (s, 3H, H_{10}), 2.39 (s, 3H, H_{14}), 1.86–1.78 (m, 2H, H_{57}), 1.34 (s, 9H, H_{1}), 1.17 (s, 9H, H_{52}), 1.04 (t, J = 7.4 Hz, 3H, H_{58}); ^{13}C NMR (100.6 MHz, $CDCl_3$): δ = 177.3 (quat. C, C_{63}), 174.6 (quat. C, C_{42}), 174.5 (quat. C, C_{43}), 165.9 (quat. C, C_{18}), 164.0 (quat. C, C_{30}), 159.9 (quat. C, C_{55}), 155.1 (quat. C, C_{11}), 152.6 (quat. C, C_{13}), 152.2 (quat. C, C_{3}), 151.2 (quat. C, C_{16}), 146.6 (quat. C, C_{47}), 146.0 (quat. C, C_{50}), 142.8 (quat. C, C_{38}), 140.7 (quat. C, C_{53}), 137.3 (CH, C_{26}), 137.0 (quat. C, C_{61}), 136.8 (quat. C, C_{35}), 136.6 (quat. C, C_{32}), 135.6 (quat. C), 133.8 (quat. C, C_{19}), 132.3 (CH, C_{27} or C_{28}) 131.7 (CH, C_{5}), 131.5 (CH, C_{21}), 129.1 (CH, C_{39}), 128.7 (quat. C, C_{41}), 128.7 (CH, C_{34}), 128.5 (CH, C_{20}), 128.4 (CH, C_{27} or C_{28}), 126.5 (CH, C_{49}), 126.3 (CH, C_{40}), 126.2 (quat. C, C_{22}), 125.6 (CH, C_{4}), 125.2 (quat. C), 123.4 (quat. C), 121.3 (CH, C_{12}), 120.5 (CH, C_{33}), 120.0 (CH, C_{60}), 119.8 (quat. C, C_{6}), 115.9 (CH, C_{59}),

114.0 (CH, C_{15}), 113.8 (CH, C_{48}), 110.6 (CH, C_{54}), 91.6 (quat. C, C_{7}), 90.8 (quat. C, C_{8} or C_{24}), 89.6 (quat. C, C_{23}), 87.2 (quat. C, C_{8} or C_{24}), 77.7 (CH, C_{44}), 69.9 (CH_2, C_{56}), 69.3 (CH, C_{46}), 58.2 (CH, C_{45}), 41.6 (CH_2, C_{62}), 37.6 (CH_2, C_{37}), 37.0 (CH_2, C_{36}), 35.0 (quat. C, C_{2}), 34.2 (quat. C, C_{51}), 31.5 (CH_3, C_{52}), 31.3 (CH_3, C_{1}), 22.7 (CH_2, C_{57}), 22.0 (CH_3, C_{10}), 21.7 (CH_3, C_{14}), 10.7 (CH_3, C_{58}); MS (ES+): *m/z* (%) = 1170 ($[M+H]^+$, 100), 1171 (20); HRMS (MALDI): *m/z* calc. for $[M+H]^+$ $C_{75}H_{72}N_5O_8$ 1170.5381, found 1170.5251.

2.4 Synthesis of recognition-disabled nitrone S_{dis}

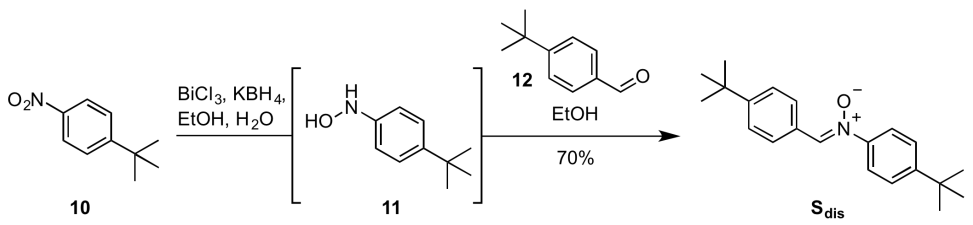

***N*-(4-*tert*-butylphenyl)-*N*-[(1Z)-(4-*tert*-butylphenyl)methylene]amine oxide S_{dis}.**

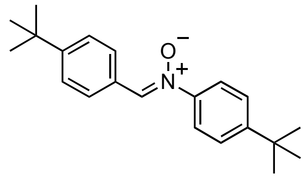

$BiCl_3$ (224 mg, 0.71 mmol) was added to a solution of 1-*tert*-butyl-4-nitrobenzene **10** (0.6 mL, 3.54 mmol) in EtOH (18 mL) and water (6 mL). KBH_4 (383 mg, 7.10 mmol) was then added gradually over 3 min under a N_2 atmosphere. After the addition was complete, the reaction mixture was stirred at ambient temperature for a further 13 min under a N_2 atmosphere. Then the mixture was acidified to pH 7 with 0.5 M HCl under a N_2 atmosphere and extracted immediately with Et_2O (2 ×). The organic layers were combined, washed with saturated NaCl solution, dried (Na_2SO_4) and concentrated by evaporation under reduced pressure. The 4-*tert*-butyl-*N*-hydroxyaniline **11** obtained was dissolved immediately in EtOH (10 mL) and 4-*tert*-butylbenzaldehyde **12** (0.6 mL, 3.59 mmol) was added. The reaction mixture was stirred at ambient temperature in the absence of light for 23 h. The solvent was removed by evaporation under reduced pressure. The residue was purified by column chromatography (SiO_2, gradient from 99.5:0:0.5 to 94.5:5.0:0.5 cyclohexane/EtOAc/Et_3N) to give $\mathbf{S_{dis}}$ (767 mg, 70% over 2 steps) as a pale yellow solid. ^1H NMR (300.1 MHz, $CDCl_3$): δ = 8.35–8.30 (m, AA' of AA'BB' system, 2H, Ar CH), 7.88 (s, 1H, CH), 7.72–7.67 (m, AA' of AA'BB' system, 2H, Ar CH), 7.52–7.46 (m, BB' of AA'BB' system, 4H, Ar CH), 1.35 (s, 18H, CH_3); ^{13}C NMR (75.5 MHz, $CDCl_3$): δ = 154.5 (quat. C), 153.3 (quat. C), 146.8 (quat. C), 134.2 (CH), 129.1 (CH), 128.3 (quat. C), 126.1 (CH), 125.7 (CH),

121.4 (CH), 35.2 (quat. C), 35.0 (quat. C), 31.4 (CH_3), 31.3 (CH_3); MS (ES+): *m/z* (%) = 332 ($[M+Na]^+$, 95), 641 (100); HRMS (ES+): *m/z* calc. for $[M+Na]^+$ $C_{21}H_{27}NONa$ 332.1990, found 332.1994.

3. Kinetic studies

3.1 ^{1}H NMR spectroscopic binding study between M and L

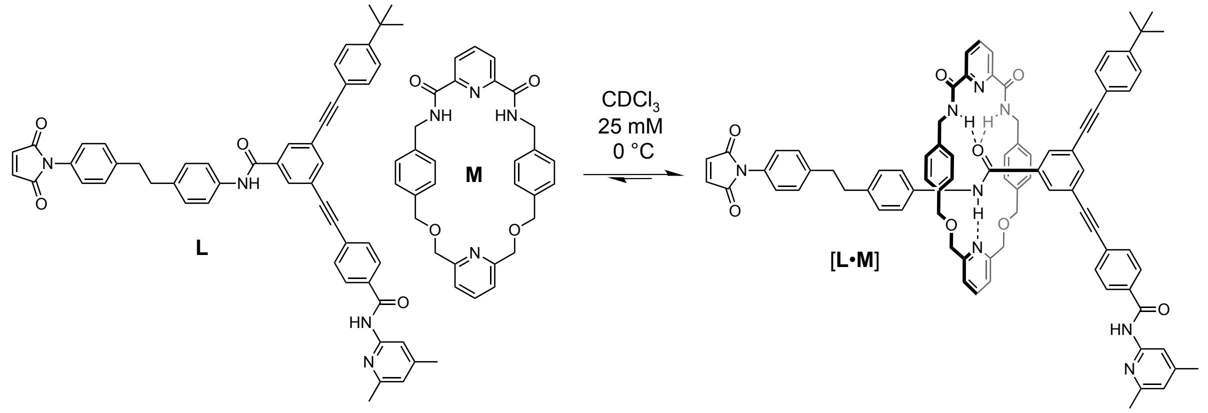

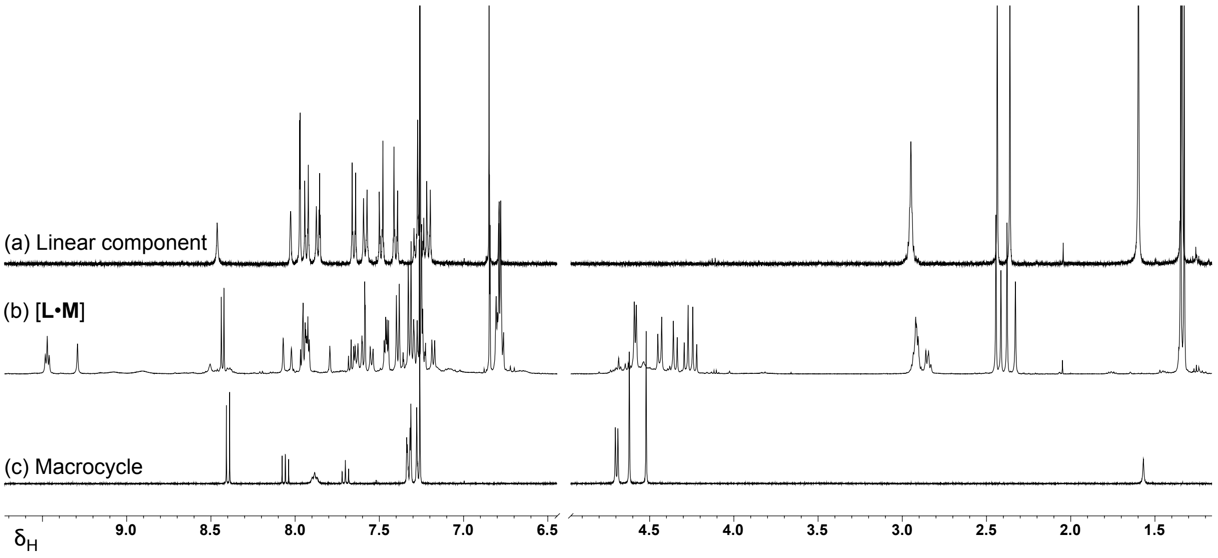

Fig. S1 Partial ^{1}H NMR spectrum of (a) linear component **L** (400.1 MHz, 21 °C, $CDCl_3$), (b) equimolar mixture (25 mM) of linear compound **L** and macrocycle **M** (499.9 MHz, 0 °C, $CDCl_3$), (c) **M** (400.1 MHz, 21 °C, $CDCl_3$).

3.2 Thread recognition-disabled kinetic experiments

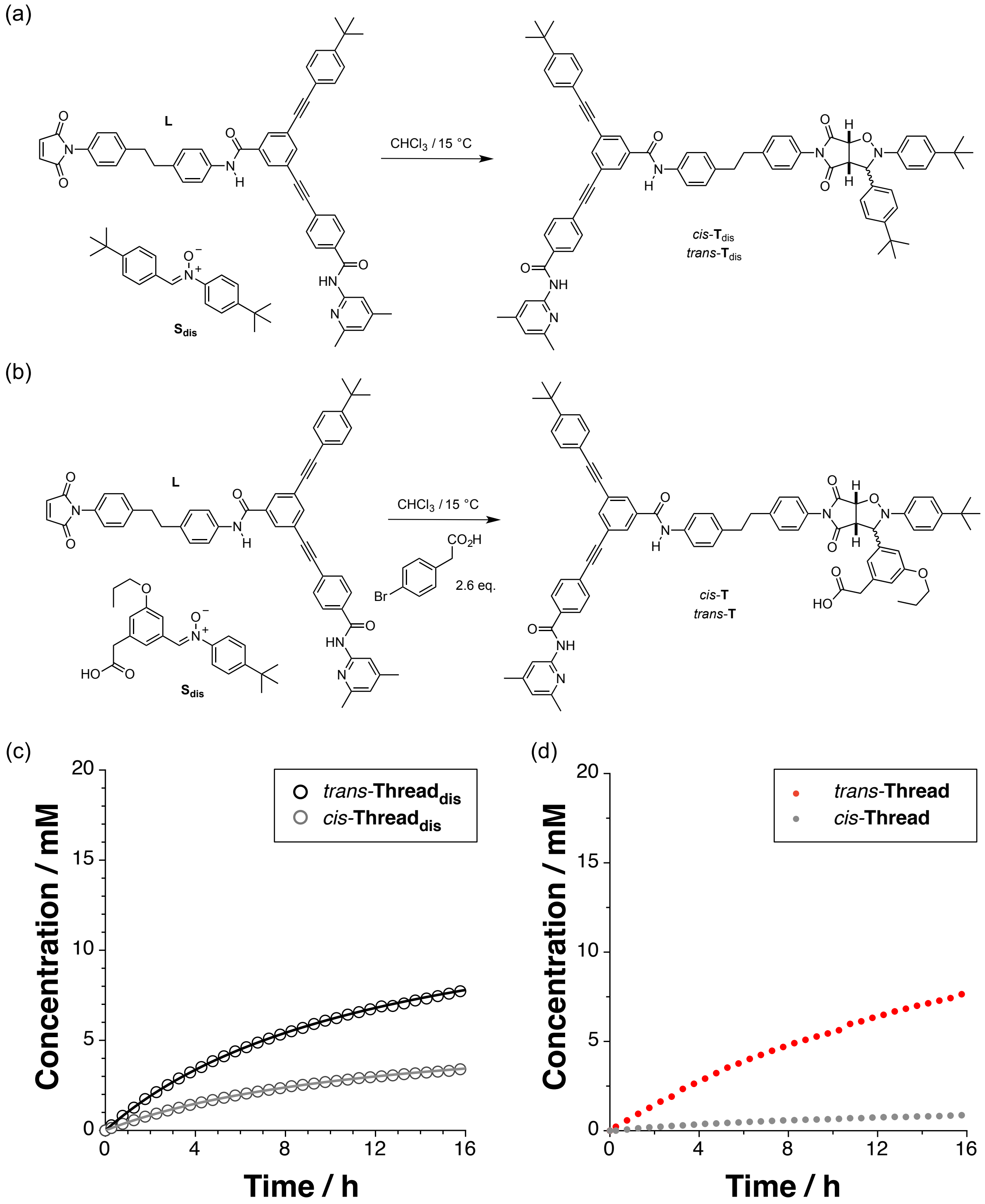

Fig. S2 (a) Reaction scheme and the (c) concentration *vs.* time profile for the reaction between recognition-disabled nitrone $\mathbf{S_{dis}}$ and maleimide **L** (15 °C, $CDCl_3$, 20 mM), resulting in the formation of recognition-disabled *trans*-$\mathbf{T_{dis}}$ (empty black circles) and *cis*-$\mathbf{T_{dis}}$ cycloadduct (empty grey circles), as determined by ^{1}H NMR spectroscopy (500 MHz). Black and grey lines represent the results of kinetic fitting determined using SimFit simulation and fitting software (for *trans*- and *cis*-**T**, respectively). (b) Reaction scheme and (d) concentration *vs.* time profile for the reaction between nitrone **S** and maleimide **L** (15 °C, $CDCl_3$, 20 mM) in the presence of 2.6 eq. of 4-bromophenylacetic acid as an inhibitor, affording the *trans*-**T** cycloadduct (filled red circles) and *cis*-**T** cycloadduct (filled grey circles), as determined by ^{1}H NMR spectroscopy (500 MHz).

3.3 Rotaxane recognition-disabled kinetic experiment

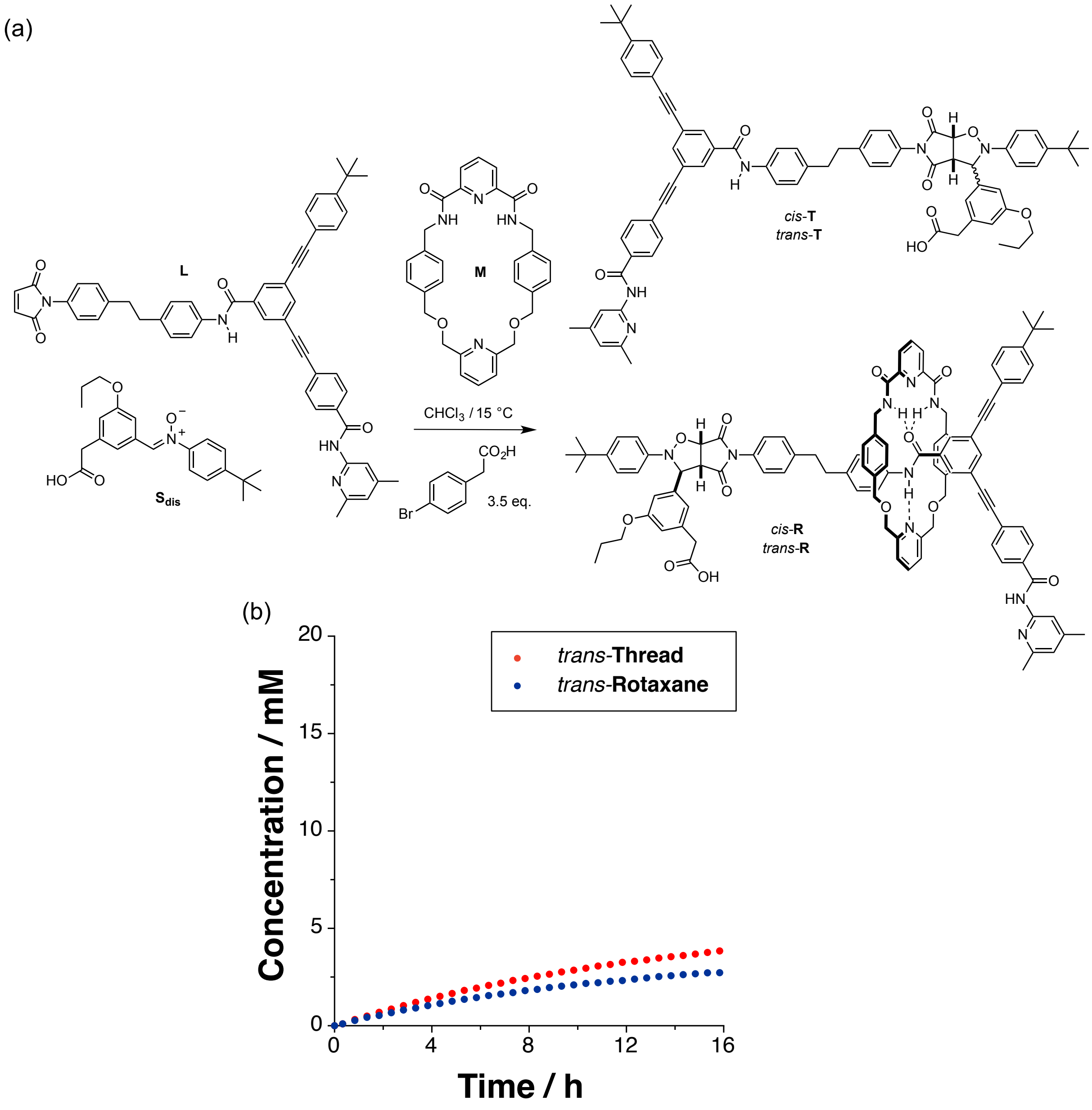

Fig. S3 (a) Reaction scheme and (b) concentration *vs.* time profile for the reaction between maleimide **L**, macrocycle **M** and nitrone **S** (15 °C, $CDCl_3$, 20 mM) in the presence of 3.5 eq. of 4-bromophenylacetic acid as an inhibitor. The formation of rotaxane *trans*-**R** is shown as dark blue filled circles and the formation of thread *trans*-**T** as dark red filled circles (as determined by 500 MHz ^{1}H NMR spectroscopy). Formation of *cis* products could not be accurately followed at all reaction time points during this kinetic experiment and is therefore not shown.

4. Example SimFit fitting scripts

4.1 SimFit input file for the bimolecular reaction of L with recognition-disabled nitrone S_{dis}

```
*=============================================
* Formation of thread through bimolecular pathway, recognition disabled
*=============================================
* A = M
* B = Nitrone (recognition disabled)
*=============================================
DIM ( 2 )

* Bimolecular routes to TRANS and CIS

REACTION ( A + B --> TRANS )  CONSTANT ( 1, 1.84E-4, 1, 1, 100 )
REACTION ( A + B --> CIS   )  CONSTANT ( 2, 1.84E-4, 2, 1, 100 )

REACTION (COMPILE)
REACTION (SHOW)
CONSTANT (SHOW)

DEFINE   ( 1, TRANS , P, 1) SCALE (3,1)
DEFINE   ( 2, CIS   , P, 3) SCALE (3,1)

SELECT   ( TRANS, CIS )
READ     ( THREADBI )
REACTION ( DOC  )
CONSTANT ( DOC  )

TIME     (SEC)
WIN      (0, 60000, 15000, 200, 0, 20E-3, 20E-3, 1E-4)

ASSIGN   (OBS,  TRANS = TRANS  )
ASSIGN   (OBS,  CIS   = CIS    )
ASSIGN   (SPEC, A     = #20e-3 )
ASSIGN   (SPEC, B     = #20e-3 )

CHOOSE   ( EXP1   )
INTEG    ( STIFF, 1E-9, 4, 0.05, 200, 100 )
PLOT     (OBS, RES)

OPAR     (1E16)

SIMPLEX  (PLOT)
SIMPLEX  (PLOT)
SIMPLEX  (PLOT)
SIMPLEX  (PLOT)
NEWTON   (PLOT)

PLOT     (FILE)
```

4.2 SimFit input file for thread forming reaction of L with S

```
*********************************************************************************
* Thread kinetics
* Reaction between S and L to make T
*********************************************************************************
* Ka for individual binding event is 250 M-1, hence K off 4.0 x 10^6
* L  = linear component and S = stopper
* T  = Thread and R = Rotaxane
* Three experiments are fitted simultaneously:
* Native
* Doped with T
* Doped with R
*********************************************************************************

DIM (2)
* Bimolecular routes to T
REACTION ( L + S   --> T  ) CONSTANT ( 1, 7.7275E-4, 0 )

* Formation of binary complexes including product duplexes
REACTION ( L + T  ==> LT ) CONSTANT (  2, 1E9, 0) CONSTANT (  3, 4.0E+6, 0)
REACTION ( S + T  ==> ST ) CONSTANT (  4, 1E9, 0) CONSTANT (  5, 4.0E+6, 0)
REACTION ( L + ST ==> LST) CONSTANT (  6, 1E9, 0) CONSTANT (  7, 4.0E+6, 0)
REACTION ( S + LT ==> LST) CONSTANT (  8, 1E9, 0) CONSTANT (  9, 4.0E+6, 0)
REACTION ( T + T  ==> TT ) CONSTANT ( 10, 1E9, 0) CONSTANT ( 11, 4.0E+3, 1, 1, 1000 )
REACTION ( L + S  ==> LS ) CONSTANT ( 12, 1E9, 0) CONSTANT ( 13, 4.0E+6, 0)

* Ternary complex reaction
REACTION ( LST    --> TT ) CONSTANT ( 14, 1E-4, 2, 1, 100 )

*Bimolecular Reactions of Complexes
REACTION ( LS + L           --> T  + L          )  CONSTANT ( 15, 7.7275E-4, 0 )
REACTION ( LS + S           --> T  + S          )  CONSTANT ( 16, 7.7275E-4, 0 )
REACTION ( LS + LT          --> T  + L + T      )  CONSTANT ( 17, 7.7275E-4, 0 )
REACTION ( LS + ST          --> T  + S + T      )  CONSTANT ( 18, 7.7275E-4, 0 )
REACTION ( LS + LST         --> T  + L + S + T  )  CONSTANT ( 19, 7.7275E-4, 0 )
REACTION ( 2 LS             --> T  + L + S      )  CONSTANT ( 20, 7.7275E-4, 0 )

* reactions with R  template
* Formation of binary complexes including product duplexes with R
REACTION ( L + R  ==> LR   )  CONSTANT ( 21, 1E9, 0) CONSTANT ( 22, 4.0E+6, 0 )
REACTION ( S + R  ==> SR   )  CONSTANT ( 23, 1E9, 0) CONSTANT ( 24, 4.0E+6, 0 )
REACTION ( L + SR ==> LSR  )  CONSTANT ( 25, 1E9, 0) CONSTANT ( 26, 4.0E+6, 0 )
REACTION ( S + LR ==> LSR  )  CONSTANT ( 27, 1E9, 0) CONSTANT ( 28, 4.0E+6, 0 )
REACTION ( R  + R ==> RR   )  CONSTANT ( 29, 1E9, 0) CONSTANT ( 30, 4.0E+6, 0 )
REACTION ( R  + T ==> TR   )  CONSTANT ( 31, 1E9, 0) CONSTANT ( 32, 4.0E+6, 0 )

*Bimolecular Reactions of Complexes
REACTION ( LS + LR          --> T  + L + R      )  CONSTANT ( 33, 7.7275E-4, 0 )
REACTION ( LS + SR          --> T  + S + R      )  CONSTANT ( 34, 7.7275E-4, 0 )
REACTION ( LS + LSR         --> T  + L + S + R  )  CONSTANT ( 35, 7.7275E-4, 0 )

*Binary complex reactivity to make Thread
REACTION ( LS                --> T               )  CONSTANT ( 36, 1.5455E-5, 0 )

REACTION ( COMPILE )
REACTION ( SHOW )
CONSTANT ( SHOW )

DEFINE   ( 1, T , P, 2 ) SCALE (3,1)
DEFINE   ( 2, R , P, 4 ) SCALE (3,1)
SELECT   ( T , R )

READ     ( THREAD )

REACTION ( DOC )
CONSTANT ( DOC )
TIME     ( SEC )

WIN      ( 0, 60000, 20000, 50, 0, 25e-3, 5e-3, 1e-4 )

ASSIGN   ( OBS,  L = L +   LS + LT  + LST  + LR   + LSR  )
ASSIGN   ( OBS,  S = S +   LS + ST  + LST  + SR   + LSR  )
ASSIGN   ( OBS,  T = T +   LT + ST  + LST  + 2 TT + TR   )
ASSIGN   ( OBS,  R = R + 2 RR + LR  + SR   + LSR  + TR   )

ASSIGN   ( SPEC, L = #20E-3 )
ASSIGN   ( SPEC, S = #20E-3 )
ASSIGN   ( SPEC, T = T      )
ASSIGN   ( SPEC, R = R      )

CHOOSE   ( EXP1, EXP2, EXP3 )
```

```
OPAR     ( 1E20 )
INTEG    (STIFF, 1E-10, 50, 0.025, 100, 50)
PLOT     (OBS, RES)

* Optimise rate constant using simplex

SIMPLEX  (PLOT)
SIMPLEX  (PLOT)
NEWTON   (PLOT)
PLOT     (FILE)
PLOT     (OBS, RES)
```

4.3 SimFit input file for thread and rotaxane forming reaction of L with S in the presence of M

```
***************************************************************************************
* Rotaxane kinetics
* Reaction between stopper S with M and L to make T and R
***************************************************************************************
* Ka for individual binding event is 250 M-1
* L = linear component and S = stopper
* T = Thread and R = Rotaxane
* Three experiments are fitted simultaneously:
* Native
* Doped with T
* Doped with R
***************************************************************************************
DIM (2 )

* Bimolecular routes to T
REACTION ( L + S            --> T       )   CONSTANT (  1, 7.7275E-4, 0 )

* Formation of binary complexes including product duplexes
REACTION ( L      + T  ==> LT       )  CONSTANT ( 2, 1E9, 0) CONSTANT (  3, 4.0E+6, 0)
REACTION ( S      + T  ==> ST       )  CONSTANT ( 4, 1E9, 0) CONSTANT (  5, 4.0E+6, 0)
REACTION ( L      + ST ==> LST      )  CONSTANT ( 6, 1E9, 0) CONSTANT (  7, 4.0E+6, 0)
REACTION ( S      + LT ==> LST      )  CONSTANT ( 8, 1E9, 0) CONSTANT (  9, 4.0E+6, 0)
REACTION ( T      + T  ==> TT       )  CONSTANT (10, 1E9, 0) CONSTANT ( 11,2.068E+4,0)
REACTION ( L      + S  ==> LS       )  CONSTANT (12, 1E9, 0) CONSTANT ( 13, 4.0E+6, 0)

* Ternary complex reaction
REACTION ( LST              --> TT  )  CONSTANT ( 14, 3.6225E-4 , 0 )

* Bimolecular Reactions of Complexes
REACTION ( LS + L           --> T  + L          )  CONSTANT ( 15, 7.7275E-4, 0 )
REACTION ( LS + S           --> T  + S          )  CONSTANT ( 16, 7.7275E-4, 0 )
REACTION ( LS + LT          --> T  + L + T      )  CONSTANT ( 17, 7.7275E-4, 0 )
REACTION ( LS + ST          --> T  + S + T      )  CONSTANT ( 18, 7.7275E-4, 0 )
REACTION ( LS + LST         --> T  + L + S + T  )  CONSTANT ( 19, 7.7275E-4, 0 )
REACTION ( 2 LS             --> T  + L + S      )  CONSTANT ( 20, 7.7275E-4, 0 )

* Reactions with rotaxane template
* Formation of binary complexes including product duplexes with R
REACTION ( L + R        ==> LR )  CONSTANT ( 21, 1E9, 0 ) CONSTANT ( 22, 4.0E+6, 0)
REACTION ( S + R        ==> SR )  CONSTANT ( 23, 1E9, 0 ) CONSTANT ( 24, 4.0E+6, 0)
REACTION ( L + SR       ==> LSR)  CONSTANT ( 25, 1E9, 0 ) CONSTANT ( 26, 4.0E+6, 0)
REACTION ( S + LR       ==> LSR)  CONSTANT ( 27, 1E9, 0 ) CONSTANT ( 28, 4.0E+6, 0)
REACTION ( R  + R       ==> RR )  CONSTANT ( 29, 1E9, 0 ) CONSTANT ( 30, 4.0E+6, 0)
REACTION ( R  + T       ==> TR )  CONSTANT ( 31, 1E9, 0 ) CONSTANT ( 32, 4.0E+6, 0)

* Bimolecular Reactions of Complexes
REACTION ( LS + LR          --> T + L + R       )  CONSTANT ( 33, 7.7275E-4, 0 )
REACTION ( LS + SR          --> T + S + R       )  CONSTANT ( 34, 7.7275E-4, 0 )
REACTION ( LS + LSR         --> T + L + S + R   )  CONSTANT ( 35, 7.7275E-4, 0 )

* Binary complex reactivity to make T
REACTION ( LS         --> T               )  CONSTANT ( 36, 1.5455E-5, 0 )

* Reactions involving rotaxane template
* Formation of binary complexes including product duplexes with rotaxane
REACTION ( L + R   ==> LR      )  CONSTANT ( 37, 1E9, 0 ) CONSTANT ( 38, 4.0E+6, 0)
REACTION ( S + R   ==> SR      )  CONSTANT ( 39, 1E9, 0 ) CONSTANT ( 40, 4.0E+6, 0)
REACTION ( L + SR  ==> LSR     )  CONSTANT ( 41, 1E9, 0 ) CONSTANT ( 42, 4.0E+6, 0)
REACTION ( S + LR  ==> LSR     )  CONSTANT ( 43, 1E9, 0 ) CONSTANT ( 44, 4.0E+6, 0)

* Bimolecular Reactions of Complexes
REACTION ( LS + LR          --> T  + L + R      )  CONSTANT ( 45, 7.7275E-4, 0 )
REACTION ( LS + SR          --> T  + S + R      )  CONSTANT ( 46, 7.7275E-4, 0 )
REACTION ( LS + LSR         --> T  + L + S + R  )  CONSTANT ( 47, 7.7275E-4, 0 )

* Added to account for rotaxane formation
REACTION ( L   + M   ==> LM  )  CONSTANT ( 48, 1E9, 0 ) CONSTANT (49,5.00E+6,1,1, 100)
REACTION ( LM  + S   ==> LMS )  CONSTANT ( 50, 1E9, 0 ) CONSTANT ( 51, 4.0E+6, 0)
REACTION ( LMS       --> R   )  CONSTANT ( 52, 1E-5, 2, 1, 100 )
REACTION ( LM + S    --> R   )  CONSTANT ( 53, 5.8E-4, 0 )
REACTION ( LM  + T   ==> LMT )  CONSTANT ( 54, 1E9, 0 ) CONSTANT ( 55, 4.0E+6, 0)
REACTION ( LM  + R   ==> LMR )  CONSTANT ( 56, 1E9, 0 ) CONSTANT ( 57, 4.0E+6, 0)
REACTION ( S   + LMR ==> LMSR)  CONSTANT ( 58, 1E9, 0 ) CONSTANT ( 59, 4.0E+6, 0)
REACTION ( S   + LMT ==> LMST)  CONSTANT ( 60, 1E9, 0 ) CONSTANT ( 61, 4.0E+6, 0)
REACTION ( LM   + SR ==> LMSR)  CONSTANT ( 62, 1E9, 0 ) CONSTANT ( 63, 4.0E+6, 0)
REACTION ( LM   + ST ==> LMST)  CONSTANT ( 64, 1E9, 0 ) CONSTANT ( 65, 4.0E+6, 0)
REACTION ( LM + ST   --> T  + R)CONSTANT ( 66, 5.8E-4, 0 )
REACTION ( S  + LMT  --> T  + R)CONSTANT ( 67, 5.8E-4, 0 )
REACTION ( LM + SR   --> R  + R)CONSTANT ( 68, 5.8E-4, 0 )
REACTION ( S  + LMR--> R  + R)  CONSTANT ( 69, 5.8E-4, 0 )
```

```
REACTION ( LS + M  ==> LMS    )  CONSTANT ( 70, 1E9, 0 ) CONSTANT (71,5.00E+6,1,1,100)
REACTION ( M  + LSR--> R  + R)  CONSTANT ( 72, 5.8E-4, 0 )
REACTION ( M  + LST--> R  + T)  CONSTANT ( 73, 5.8E-4, 0 )

REACTION ( COMPILE )
REACTION ( SHOW )
CONSTANT ( SHOW )

DEFINE   ( 1, T , P, 2 ) SCALE (3,1)
DEFINE   ( 2, R , P, 4 ) SCALE (3,1)
SELECT   ( T , R )

READ     ( ROTAXANE )
REACTION ( DOC )
CONSTANT ( DOC )
TIME     ( SEC )

WIN     (0, 60000, 20000, 50, 0, 25e-3, 5e-3, 1e-4 )
ASSIGN  (OBS, L = L + LS + LT  + LST  + LR   + LSR + LMT + LMR  + LMS + LMST + LMSR  )
ASSIGN  (OBS, S = S + LS + ST  + LST  + SR   + LSR + LMS + LMST + LMSR               )
ASSIGN  (OBS, T = T + LT + ST  + LST  + 2 TT + TR  + LMT + LMST                      )
ASSIGN  (OBS, R = R + 2 RR + LR  + SR   + LSR  + TR  + LMR + LMSR                    )

ASSIGN   ( SPEC, L = #20E-3 )
ASSIGN   ( SPEC, S = #20E-3 )
ASSIGN   ( SPEC, M = #20E-3 )
ASSIGN   ( SPEC, T = T      )
ASSIGN   ( SPEC, R = R      )

CHOOSE   ( EXP1, EXP2, EXP3 )
OPAR     ( 1E23 )
INTEG    ( STIFF, 1E-13, 50, 0.025, 100, 50 )
PLOT     ( OBS, RES )

* Optimise rate constant using simplex

SIMPLEX  (PLOT)
SIMPLEX  (PLOT)
SIMPLEX  (PLOT)

NEWTON   (PLOT)
PLOT     (FILE)
PLOT     (OBS, RES)
```

5. References

1. M. W. Schmidt, K. K. Baldridge, J. A. Boatz, S. T. Elbert, M. S. Gordon, J. H. Jensen, S. Koseki, N. Matsunaga, K. A. Nguyen, S. J. Su, T. L. Windus, M. Dupuis, J. A. Montgomery, *J. Comput. Chem.,* 1993, **14**, 1347.
2. M. S. Gordon, M. W. Schmidt, *Advances in electronic structure theory: GAMESS a decade later* in *Theory and Applications of Computational Chemistry, the First Forty Years*, Chapter 41, 1167-1189, C. E. Dykstra, G. Frenking, K. S. Kim, G. E. Scuseria, Eds. Elsevier, Amsterdam, 2005.
3. G. B. Rocha, R. O. Freire, A. M. Simas, J. J. P. Stewart, *J. Comput. Chem.,* 2006, **27**, 1101.
4. T. Kosikova, N. I. Hassan, D. B. Cordes, A. M. Z. Slawin and D. Philp, *J. Am. Chem. Soc.,* asap, DOI: 10.1021/jacs.5b09738.
5. A. Vidonne and D. Philp, *Tetrahedron*, 2008, **64**, 8464.

6. XYZ Files for computed transition state geometries

6.1 Computed transition state geometry for trans-T formed through binary complex

```
 159
scf done:  -513.527224
 C    5.117978   -1.655753    0.289204
 C    4.548861   -3.021636    0.432961
 N    3.709895   -3.182081    1.532455
 C    3.013354   -4.376689    1.844910
 C    2.509530   -5.217442    0.850297
 H    2.654888   -5.002891   -0.212694
 C    1.790319   -6.348859    1.214769
 C    1.540761   -6.637329    2.551924
 C    0.745800   -7.839804    2.938077
 C   -0.505836   -8.023692    2.076059
 H   -0.244612   -8.509191    1.111572
 H   -1.194370   -8.752953    2.557377
 C   -1.220403   -6.730894    1.864198
 C   -1.690160   -6.012250    2.960251
 H   -1.544896   -6.398612    3.973662
 C   -2.340769   -4.798940    2.785435
 C   -2.540931   -4.312588    1.490396
 C   -2.099032   -5.038397    0.381958
 H   -2.260297   -4.677193   -0.638041
 C   -1.433708   -6.240832    0.579419
 H   -1.079016   -6.798100   -0.293533
 N   -3.194584   -3.064866    1.301001
 C   -4.344112   -2.619056    2.026018
 O   -4.896196   -3.255240    2.898935
 C   -4.674736   -1.259984    1.496402
 O   -6.261453   -1.819875    0.356139
 N   -5.762637   -2.107184   -0.752543
 C   -5.716263   -3.508179   -1.158798
 C   -5.960954   -4.525732   -0.236736
 C   -5.955335   -5.843869   -0.669684
 H   -6.139191   -6.625028    0.075815
 C   -5.720509   -6.168026   -2.007177
 C   -5.711889   -7.621706   -2.403060
 C   -7.088856   -8.219580   -2.102829
 H   -7.153274   -9.273718   -2.410209
 H   -7.346338   -8.199574   -1.034883
 H   -7.890779   -7.684090   -2.630881
 C   -4.634185   -8.346181   -1.591613
 H   -4.830620   -8.345180   -0.510532
 H   -4.544751   -9.403680   -1.880649
 H   -3.642835   -7.891896   -1.732265
 C   -5.412010   -7.839859   -3.886760
 H   -5.401131   -8.909462   -4.146860
 H   -6.164992   -7.379993   -4.542710
 H   -4.427259   -7.448337   -4.179512
 C   -5.513658   -5.134348   -2.916817
 H   -5.346309   -5.326115   -3.980575
 C   -5.519248   -3.806733   -2.506061
 H   -5.388113   -3.021776   -3.258174
 H   -6.159551   -4.303375    0.818902
 C   -4.869543   -1.202938   -1.301694
 C   -3.757552   -0.937882    0.496989
 C   -2.810608   -2.079913    0.340990
 O   -1.881658   -2.189757   -0.433563
 H   -3.431807    0.072502    0.247998
 H   -4.137714   -1.597377   -2.031115
 C   -5.231809    0.210236   -1.488200
 C   -6.501791    0.705002   -1.205284
 H   -7.284396    0.048602   -0.817696
 C   -6.760678    2.054400   -1.449881
 O   -7.966071    2.658464   -1.254000
 C   -8.856440    2.041403   -0.342781
 C   -9.914962    3.113007   -0.056366
 C  -10.956772    2.557085    0.898774
 H  -11.474201    1.677467    0.488987
 H  -10.522191    2.251380    1.861622
 H  -11.733993    3.300922    1.126788
 H  -10.390304    3.454181   -1.000268
 H   -9.441512    4.024619    0.365337
 H   -9.314822    1.134520   -0.797485
 H   -8.348027    1.716869    0.593208
```

C	-5.789136	2.905176	-1.987855
H	-6.021836	3.957272	-2.185196
C	-4.536035	2.381838	-2.276419
C	-4.249108	1.045211	-2.026602
H	-3.252221	0.649823	-2.249282
C	-3.500378	3.275873	-2.875534
H	-3.854742	3.676386	-3.851111
H	-2.554953	2.742468	-3.113472
C	-3.205945	4.376642	-1.893881
O	-2.431910	4.329547	-0.950058
O	-3.901009	5.499857	-2.138296
H	-3.702437	6.201709	-1.494099
H	-5.294334	-0.567630	2.066604
H	-2.685551	-4.242139	3.662030
H	1.393119	-8.741464	2.865974
H	0.458401	-7.799096	4.011044
C	2.053500	-5.797392	3.538100
H	1.878357	-6.014123	4.596679
C	2.792291	-4.676281	3.196018
H	3.190628	-4.035714	3.988776
H	1.416383	-7.007950	0.425428
H	3.621221	-2.421345	2.199384
O	4.815240	-3.925747	-0.347855
C	4.296116	-0.537220	0.352473
C	4.864655	0.730075	0.212370
C	4.043758	1.869092	0.310005
C	3.348711	2.820749	0.409442
C	2.511907	3.945850	0.536878
C	1.327901	3.841848	1.268831
C	0.505749	4.952137	1.408208
H	-0.416053	4.871152	1.993798
C	0.870463	6.154938	0.812928
C	0.037184	7.371907	0.974055
N	-1.328692	7.178869	0.743174
H	-1.635108	6.291978	0.337094
C	-2.298799	8.203379	0.847851
N	-3.376385	8.037356	0.011964
C	-4.351157	8.969938	0.089232
C	-5.530378	8.795768	-0.811596
H	-5.471955	7.909507	-1.461622
H	-6.458619	8.700512	-0.227795
H	-5.649525	9.666657	-1.474128
C	-4.304888	10.072537	0.951934
H	-5.119067	10.799935	0.955962
C	-3.208359	10.208180	1.793152
C	-3.104023	11.350359	2.738045
H	-3.982881	12.011116	2.727092
H	-2.980400	11.005614	3.776274
H	-2.230749	11.979370	2.504854
C	-2.186875	9.260483	1.761343
H	-1.339085	9.358141	2.444021
O	0.518070	8.454755	1.280546
C	2.052231	6.263636	0.085484
C	2.875847	5.155345	-0.056880
H	3.806018	5.230130	-0.627649
H	2.334346	7.221299	-0.365543
H	1.048340	2.890054	1.730140
C	6.233885	0.877249	-0.007523
H	6.673154	1.873419	-0.118240
C	7.036412	-0.260794	-0.087058
C	6.483266	-1.534688	0.059493
H	7.115325	-2.426644	-0.012642
C	8.417498	-0.124707	-0.315804
C	9.578863	-0.012050	-0.509194
C	10.960897	0.120915	-0.738271
C	11.754024	-1.012604	-0.898708
H	11.302761	-2.008216	-0.847703
C	13.118883	-0.877438	-1.124535
H	13.699964	-1.795666	-1.244451
C	13.708357	0.381924	-1.193279
C	12.901534	1.510738	-1.031198
C	11.538980	1.388754	-0.805390
H	10.918634	2.281108	-0.680325
H	13.327033	2.517932	-1.078953
C	15.181590	0.583751	-1.435661
C	15.952184	-0.728041	-1.593364
H	15.600763	-1.323384	-2.448206
H	15.893534	-1.361754	-0.696905
H	17.024624	-0.552625	-1.768915

C	15.362871	1.395674	-2.721130
H	14.891896	0.903269	-3.583893
H	14.932556	2.404752	-2.660677
H	16.424517	1.534325	-2.973121
C	15.777906	1.341686	-0.246327
H	15.359776	2.349951	-0.121250
H	15.609233	0.810566	0.701337
H	16.864881	1.475816	-0.347918
H	3.214462	-0.636478	0.491640

6.2 Computed transition state geometry for trans-T formed through ternary complex

318
scf done: -1027.086669

C	12.543397	9.546528	0.771806
C	12.732096	8.258730	1.299108
N	13.421475	8.035587	2.468917
C	13.942000	9.116258	3.102890
C	13.817595	10.419831	2.617041
C	13.107290	10.628464	1.439127
N	12.193376	7.103051	0.695632
C	12.056655	6.957624	-0.688958
O	12.371742	7.823687	-1.494728
C	14.671144	8.873871	4.384482
C	12.931712	11.994817	0.883155
C	11.481459	5.657558	-1.115583
C	12.040020	4.456534	-0.693505
C	11.490998	3.256160	-1.125934
C	10.389751	3.268921	-1.982626
C	9.841225	4.477256	-2.416253
C	10.392639	5.675073	-1.982524
C	9.827555	2.049761	-2.407149
C	9.358448	1.020994	-2.754981
C	8.791929	-0.207746	-3.143932
C	9.580801	-1.357669	-3.164707
C	9.000634	-2.573585	-3.524944
C	7.645866	-2.647935	-3.855278
C	6.878492	-1.489907	-3.819865
C	7.439140	-0.264098	-3.482888
C	5.438257	-1.586924	-4.181453
O	5.065909	-2.161184	-5.196379
C	9.787904	-3.739723	-3.549175
C	10.448180	-4.720562	-3.567082
C	11.233392	-5.889379	-3.586512
C	12.436401	-5.904599	-4.293282
C	13.205918	-7.060315	-4.307573
C	12.780599	-8.192425	-3.621902
C	11.581123	-8.173271	-2.919271
C	10.801977	-7.024113	-2.898485
C	13.628952	-9.455912	-3.641802
C	12.942825	-10.542361	-2.818779
N	4.581524	-0.974181	-3.271911
C	3.175077	-0.879249	-3.399413
C	2.432285	-0.693645	-2.221032
C	1.061421	-0.521282	-2.292673
C	0.405170	-0.553787	-3.521745
C	1.141865	-0.755222	-4.681508
C	2.523907	-0.903103	-4.634186
C	-1.070743	-0.337172	-3.570815
C	-1.812067	-1.500217	-2.906374
C	-3.251466	-1.148775	-2.718145
C	-3.590354	-0.149201	-1.809460
C	-4.914591	0.212386	-1.615203
C	-5.915691	-0.462167	-2.319529
C	-5.590304	-1.474619	-3.224795
C	-4.254704	-1.801983	-3.425675
N	-7.276708	-0.111506	-2.108906
C	-7.843318	0.251184	-0.847917
C	-9.259164	0.646266	-1.120737
C	-9.510780	0.488808	-2.482625
C	-8.254467	0.029124	-3.141523
O	-7.256495	0.192389	0.213507
O	-8.048472	-0.177263	-4.320606
C	15.001343	-9.156483	-3.045546
C	13.793427	-9.935540	-5.081082
O	-8.886160	2.645169	-1.059727
N	-8.622087	2.942757	-2.247076
C	-7.238119	3.259653	-2.589759
C	-6.859693	3.384393	-3.928249
C	-5.560568	3.763912	-4.234320
C	-4.646625	4.025535	-3.220080
C	-5.032668	3.903606	-1.891045
C	-6.327508	3.521687	-1.563066
C	-3.224356	4.442591	-3.566195
C	-2.557812	3.343468	-4.388562
C	-9.508346	2.498000	-3.212217
C	-10.948849	2.757523	-3.089481

C	-11.793484	2.167284	-4.035042
C	-13.157113	2.411352	-3.994732
C	-13.689931	3.262772	-3.032786
C	-12.848115	3.868278	-2.107682
C	-11.478222	3.611580	-2.126304
C	-13.402903	4.839662	-1.120998
C	-14.687257	4.337461	-0.518792
O	-14.906454	3.171954	-0.179840
O	-13.980803	1.819339	-4.906673
C	-13.151364	1.030332	-5.729816
C	-14.001400	0.328561	-6.785004
C	-13.103808	-0.525404	-7.675826
O	-15.603790	5.275862	-0.341842
C	-2.433034	4.667039	-2.280894
C	-3.256297	5.735461	-4.376156
N	-17.876252	4.510448	0.689812
C	-18.017588	3.400780	1.490977
C	-19.256163	3.009496	2.025382
C	-20.372149	3.796218	1.762301
C	-20.245895	4.926633	0.961115
C	-18.990049	5.240802	0.435724
N	-16.837262	2.657276	1.698850
C	-16.564367	1.995079	2.901948
O	-17.312912	2.025858	3.870262
C	-18.848770	6.446180	-0.435430
C	-21.685443	3.418670	2.345079
C	-15.279747	1.254957	2.918353
C	-14.952429	0.371759	1.895410
C	-13.762397	-0.340793	1.961671
C	-12.908177	-0.159882	3.050413
C	-13.237762	0.732257	4.071978
C	-14.431672	1.437270	4.006807
C	-11.712650	-0.900243	3.125238
C	-10.719659	-1.538697	3.191735
C	-9.555437	-2.325764	3.274857
C	-9.656620	-3.703959	3.085391
C	-8.510343	-4.491744	3.185419
C	-7.273600	-3.912502	3.473375
C	-7.195728	-2.534752	3.641171
C	-8.323173	-1.729402	3.548724
C	-5.863099	-1.955277	3.953166
O	-5.176410	-2.371495	4.878546
C	-8.601304	-5.882355	2.994149
C	-8.676088	-7.051244	2.830487
C	-8.763213	-8.442280	2.633827
C	-9.988579	-9.019766	2.298712
C	-10.067279	-10.392229	2.104413
C	-8.933331	-11.184365	2.242948
C	-7.714514	-10.605544	2.577470
C	-7.622738	-9.234244	2.774390
C	-9.026496	-12.688297	2.028459
C	-9.503131	-12.970785	0.606535
N	-5.454534	-0.940821	3.094308
C	-4.163085	-0.356834	3.154310
C	-3.558007	-0.041678	4.374051
C	-2.276720	0.493929	4.378942
C	-1.600577	0.732185	3.186715
C	-2.227506	0.458865	1.975019
C	-3.502371	-0.084407	1.949782
C	-0.201093	1.250934	3.217481
C	0.712845	0.222474	3.894280
C	2.118665	0.376572	3.415767
C	2.766805	-0.681060	2.784088
C	4.074515	-0.543311	2.339262
C	4.725592	0.678855	2.515740
C	4.088735	1.748427	3.147948
C	2.786803	1.583639	3.601647
N	6.064462	0.834117	2.045205
C	6.534125	0.308903	0.819328
C	8.047959	0.535871	0.741182
C	8.410174	1.292927	2.042128
C	7.109712	1.395953	2.819754
C	9.382555	0.325238	2.746252
N	8.977773	-0.991814	2.115868
O	8.718000	-0.699099	0.781112
C	9.968744	-2.056620	2.175081
C	9.936004	-3.085569	1.227622
C	10.775373	-4.181146	1.378431
C	11.637409	-4.266224	2.464279

C	11.658851	-3.247119	3.407271
C	10.834587	-2.137650	3.269133
C	12.551387	-5.473019	2.621890
C	12.235196	-6.182464	3.935500
C	10.785957	0.806745	2.540419
C	11.614053	0.325738	1.535052
C	12.900867	0.839885	1.396498
C	13.349943	1.849856	2.233716
C	12.505455	2.353260	3.220730
C	11.231434	1.826501	3.382674
O	5.832111	-0.191656	-0.033966
O	6.969483	1.879847	3.923011
O	13.721807	0.349002	0.424085
C	15.013452	0.829484	0.722143
C	15.999440	0.321905	-0.326118
C	17.397402	0.841826	-0.003428
C	12.968978	3.490266	4.068392
C	12.825324	4.774949	3.294071
O	13.686774	5.717417	3.639608
O	11.987526	4.983583	2.412056
C	-7.652677	-13.318256	2.238965
C	-10.017245	-13.283738	3.024713
C	14.005965	-5.011842	2.630171
C	12.329178	-6.434798	1.458106
H	6.838969	0.651528	-3.487565
H	8.985305	4.480019	-3.097499
H	9.977127	6.628870	-2.325170
H	11.972170	6.292212	1.318625
H	11.961534	9.718476	-0.138099
H	13.367314	12.072968	-0.125394
H	11.866407	12.257522	0.791293
H	13.400082	12.781458	1.492335
H	14.268173	11.254159	3.159336
H	14.010073	9.060786	5.245257
H	15.058392	7.849122	4.497611
H	15.538374	9.543492	4.489826
H	12.910084	4.443896	-0.028623
H	11.918143	2.305775	-0.788664
H	10.641427	-1.306997	-2.900532
H	9.856384	-7.007360	-2.348782
H	11.248196	-9.066102	-2.381826
H	14.149208	-7.079237	-4.861493
H	12.767926	-5.013126	-4.833428
H	7.195211	-3.603945	-4.143876
H	4.975771	-0.637370	-2.393940
H	3.064713	-1.024035	-5.576884
H	0.640641	-0.789749	-5.654363
H	-1.310810	0.630306	-3.079051
H	-1.434862	-0.206197	-4.612041
H	-1.684756	-2.424171	-3.508960
H	-1.365014	-1.758401	-1.922196
H	-4.001561	-2.587040	-4.145301
H	-6.365634	-2.011877	-3.779666
H	-10.478775	0.244578	-2.917654
H	-10.836761	4.100849	-1.386352
H	-13.543205	5.826129	-1.617593
H	-12.701568	5.042774	-0.281616
H	-16.490596	4.939325	0.096430
H	-14.767803	3.451586	-3.014034
H	-11.386320	1.512761	-4.813308
H	-9.134453	2.431670	-4.253511
H	-6.612541	3.431850	-0.507964
H	-4.313274	4.109241	-1.091860
H	-5.256637	3.854370	-5.282099
H	-7.565771	3.177855	-4.742369
H	-9.996334	0.710125	-0.319934
H	-5.154667	1.025138	-0.921483
H	-2.808414	0.360782	-1.237072
H	0.495626	-0.350677	-1.371051
H	2.921378	-0.676076	-1.240833
H	-8.255765	-0.646594	3.694867
H	-12.562369	0.869407	4.921590
H	-14.708685	2.127413	4.810929
H	-16.091674	2.712277	0.967247
H	-19.359966	2.105754	2.632909
H	-21.703096	3.620033	3.427960
H	-22.532692	3.964625	1.905223
H	-21.901354	2.346646	2.217412
H	-21.106076	5.562211	0.739714

H	-17.863114	6.555856	-0.913085
H	-19.590420	6.433668	-1.248894
H	-19.019761	7.365244	0.146728
H	-15.624261	0.227333	1.042948
H	-13.496136	-1.040356	1.164051
H	-10.625270	-4.163182	2.865342
H	-6.665028	-8.776341	3.038015
H	-6.823910	-11.231482	2.686204
H	-11.025067	-10.850546	1.841537
H	-10.879361	-8.394330	2.190615
H	-6.377840	-4.535663	3.570291
H	-6.040508	-0.685842	2.301254
H	-3.976608	-0.304679	0.986537
H	-1.713082	0.657278	1.029419
H	0.166541	1.486448	2.196127
H	-0.165638	2.225394	3.749550
H	0.677906	0.329898	4.999715
H	0.336505	-0.807264	3.712388
H	2.288580	2.413592	4.112014
H	4.597661	2.705685	3.296360
H	8.350602	1.028815	-0.213792
H	9.154363	0.215215	3.839904
H	10.569271	2.222952	4.160323
H	14.018703	3.339209	4.403816
H	12.389873	3.582532	5.013060
H	13.567202	6.621269	3.124673
H	14.359710	2.254086	2.112495
H	11.286445	-0.445244	0.830646
H	9.254822	-3.053292	0.370976
H	10.752848	-4.984444	0.635252
H	12.332450	-3.314648	4.267186
H	10.880549	-1.359303	4.035520
H	8.843947	2.303589	1.868904
H	4.573622	-1.392110	1.860993
H	2.253732	-1.636861	2.635359
H	-1.799114	0.729784	5.335107
H	-4.070791	-0.213187	5.325378
H	-12.395729	1.683750	-6.233703
H	-12.625449	0.265249	-5.105341
H	-14.757030	-0.324969	-6.281033
H	-14.527414	1.093642	-7.409477
H	-13.727049	-1.039971	-8.449637
H	-12.348178	0.128127	-8.179797
H	-12.577795	-1.290470	-7.051452
H	-1.387472	4.973656	-2.535333
H	-2.921749	5.472913	-1.677895
H	-2.409649	3.719097	-1.686938
H	-2.210735	6.042079	-4.630594
H	-3.836527	5.570909	-5.318671
H	-3.745017	6.541449	-3.773241
H	-1.512251	3.650086	-4.643001
H	-2.534424	2.395428	-3.794621
H	-3.138040	3.178902	-5.330979
H	-7.721120	-14.423868	2.081316
H	-6.926232	-12.881632	1.508490
H	-7.303193	-13.111102	3.281541
H	-10.085688	-14.389349	2.867064
H	-9.667759	-13.076683	4.067275
H	-11.024527	-12.821914	2.870357
H	-9.571674	-14.076400	0.448887
H	-10.510516	-12.508865	0.452194
H	-8.776686	-12.534161	-0.123940
H	14.677787	-5.898999	2.746052
H	14.237804	-4.491704	1.667042
H	14.168860	-4.306663	3.483453
H	13.001100	-7.321953	1.573986
H	11.262618	-6.772858	1.452085
H	12.561017	-5.914661	0.494977
H	12.907018	-7.069621	4.051382
H	12.397991	-5.477288	4.788785
H	11.168634	-6.520509	3.929381
H	15.625114	-10.085360	-3.060125
H	15.504463	-8.359924	-3.648952
H	14.880787	-8.804776	-1.990205
H	14.417096	-10.864406	-5.095758
H	12.787097	-10.155042	-5.518353
H	14.296542	-9.138868	-5.684573
H	13.566495	-11.471227	-2.833455
H	12.822269	-10.190654	-1.763438

H	11.936496	-10.761864	-3.256050
H	15.005836	1.948275	0.716241
H	15.323093	0.464025	1.733388
H	16.007061	-0.796998	-0.320130
H	15.689800	0.687350	-1.337263
H	18.120353	0.469668	-0.772074
H	17.389682	1.960727	-0.009413
H	17.707042	0.476381	1.007718

6.3 Computed transition state geometry for trans-R formed through ternary complex

225
scf done: -743.488800

C	-4.210541	0.810627	0.300549
C	-5.523399	1.252570	0.432963
C	-5.766237	2.600545	0.702275
C	-4.711222	3.506430	0.825155
C	-3.405537	3.042142	0.692274
C	-3.146426	1.688605	0.456206
H	-2.106654	1.339431	0.407298
C	-2.310558	3.925357	0.743918
C	-1.353603	4.620179	0.724427
C	-0.186618	5.402378	0.636040
C	0.830560	4.983150	-0.225663
C	1.980123	5.748931	-0.357381
C	2.115365	6.919442	0.383265
C	1.106506	7.335048	1.248562
C	-0.049144	6.575656	1.376941
H	-0.848021	6.897238	2.051274
H	1.219314	8.263483	1.819262
C	3.305675	7.790455	0.235824
N	4.533640	7.114383	0.183644
H	4.550006	6.112948	0.389450
C	5.777945	7.756622	0.002069
N	6.847842	7.121968	0.581392
C	8.072322	7.686268	0.417578
C	8.278442	8.869505	-0.293498
C	7.182132	9.489627	-0.886405
C	5.915462	8.930807	-0.756718
H	5.063545	9.407323	-1.249405
C	7.343831	10.745404	-1.663850
H	6.828589	11.584021	-1.169564
H	8.392399	11.049731	-1.794655
H	6.912620	10.657107	-2.673033
H	9.280316	9.295024	-0.383731
C	9.214364	6.963582	1.056178
H	10.172012	7.488309	0.927151
H	9.065642	6.835100	2.140545
H	9.343769	5.953994	0.634477
O	3.219780	9.009086	0.167915
H	2.760212	5.425944	-1.054793
H	0.720929	4.049461	-0.789680
H	-4.907955	4.564442	1.021591
C	-7.090606	3.052533	0.845182
C	-8.204820	3.429791	0.967825
C	-9.531132	3.876537	1.113521
C	-10.586227	2.967542	1.031453
C	-11.890581	3.414988	1.175898
C	-12.167654	4.765049	1.403713
C	-11.105793	5.661663	1.483930
C	-9.793962	5.225156	1.340375
H	-8.968427	5.940084	1.405905
H	-11.262055	6.729043	1.660668
C	-13.606831	5.185452	1.551388
C	-14.356701	4.835942	0.262926
H	-13.901872	5.314782	-0.615984
H	-14.382656	3.757278	0.055856
H	-15.406409	5.162662	0.296988
C	-14.220969	4.436006	2.737081
H	-14.247028	3.346883	2.594898
H	-13.664523	4.619282	3.667350
H	-15.261341	4.741388	2.921931
C	-13.774403	6.684769	1.803371
H	-14.833737	6.964890	1.908348
H	-13.282576	7.013899	2.729898
H	-13.381440	7.297452	0.979441
H	-12.697505	2.678858	1.106683
H	-10.383233	1.907034	0.855250
H	-6.357060	0.548283	0.334588
C	-3.979739	-0.634077	0.014989
N	-3.015739	-0.894945	-0.945435
H	-2.551583	-0.077803	-1.444096
C	-2.632808	-2.187426	-1.375063
C	-2.762434	-3.336348	-0.589449
C	-2.360004	-4.566965	-1.093842
C	-1.801599	-4.676031	-2.362077

C	-1.641733	-3.526911	-3.131185
C	-2.056679	-2.295461	-2.653101
H	-1.926058	-1.414515	-3.289581
H	-1.186730	-3.586068	-4.125076
C	-1.403289	-6.009798	-2.899233
H	-2.290104	-6.681149	-2.916055
H	-1.097464	-5.942235	-3.965133
C	-0.285868	-6.647131	-2.071248
H	-0.664902	-6.934312	-1.067455
H	0.022444	-7.613306	-2.527999
C	0.894813	-5.740360	-1.970843
C	1.625943	-5.420945	-3.111694
C	2.707346	-4.553030	-3.040937
C	3.070310	-4.018824	-1.802230
C	2.370517	-4.366852	-0.643920
C	1.277576	-5.216420	-0.739819
H	0.717124	-5.465350	0.167430
H	2.660069	-3.981336	0.338218
N	4.147558	-3.095067	-1.715762
C	4.145408	-1.939431	-0.880969
C	5.464487	-1.273224	-1.059590
C	6.317469	-1.817863	0.820432
N	6.891047	-2.991444	0.360903
O	7.486314	-2.957598	-0.736074
C	6.206429	-2.006521	-1.985320
C	5.393678	-3.189045	-2.413530
O	5.673462	-4.066355	-3.202372
H	7.040093	-1.649217	-2.590354
C	6.374521	-4.272434	0.836182
C	6.064764	-4.404358	2.188991
C	5.605255	-5.625289	2.667723
C	5.468741	-6.722639	1.821415
C	5.825450	-6.579318	0.479306
C	6.283067	-5.368674	-0.020791
H	6.569363	-5.293129	-1.076657
H	5.750495	-7.421662	-0.216802
C	4.960958	-8.061161	2.291113
C	3.709837	-8.421566	1.485361
H	2.928660	-7.653566	1.579128
H	3.905228	-8.543232	0.411044
H	3.265784	-9.370318	1.820787
C	6.054528	-9.108181	2.064065
H	6.311906	-9.243498	1.004556
H	6.986458	-8.845124	2.584518
H	5.753615	-10.100395	2.431199
C	4.588410	-8.077125	3.774248
H	4.212474	-9.062810	4.088719
H	5.445356	-7.860672	4.428019
H	3.790931	-7.360046	4.016399
H	5.363023	-5.683942	3.732808
H	6.193607	-3.576343	2.893979
C	7.091699	-0.582151	1.001485
C	8.408371	-0.431890	0.574858
C	9.052556	0.781739	0.819589
C	8.420886	1.827475	1.502594
C	7.116852	1.642700	1.940082
C	6.443552	0.455532	1.677332
H	5.402937	0.342762	1.999067
C	6.454817	2.730236	2.719430
H	7.081975	3.012989	3.593607
H	5.486198	2.411837	3.160926
C	6.207936	3.900587	1.803532
O	7.005844	4.939127	2.027972
H	6.821444	5.751584	1.420531
O	5.352117	3.945299	0.925766
H	8.947933	2.768167	1.694054
O	10.341269	1.062810	0.477931
C	10.958173	0.224990	-0.480848
H	11.171272	-0.779459	-0.050593
H	10.316427	0.068745	-1.377297
C	12.255063	0.955898	-0.848325
H	12.032234	1.968932	-1.244657
H	12.870624	1.137735	0.057915
C	13.029047	0.138084	-1.867558
H	13.302868	-0.856396	-1.486285
H	12.461062	-0.021656	-2.795564
H	13.967684	0.633748	-2.154949
H	8.928983	-1.251061	0.074569
H	5.459950	-1.933016	1.510953

H	5.560360	-0.200625	-0.884950
O	3.210964	-1.591329	-0.184458
H	3.257004	-4.300671	-3.952620
H	1.349060	-5.851246	-4.079075
H	-2.488566	-5.460497	-0.474603
H	-3.159172	-3.297347	0.429785
O	-4.640940	-1.496739	0.591259
C	-6.065843	-5.673939	0.877119
C	-6.170583	-7.058812	1.064380
C	-5.299244	-7.665994	1.958981
C	-4.361472	-6.893775	2.635221
C	-4.330483	-5.516271	2.383873
N	-5.166546	-4.895016	1.518347
C	-3.317052	-4.681396	3.113641
N	-3.569091	-3.336086	3.253913
H	-4.312003	-2.881805	2.723718
C	-2.716793	-2.478207	4.082215
H	-3.384938	-1.851896	4.722758
H	-2.112195	-3.073980	4.809166
C	-1.789242	-1.625088	3.263666
C	-0.834036	-2.222240	2.447639
C	0.067743	-1.435584	1.739621
C	0.015296	-0.049286	1.845331
C	-0.946143	0.546983	2.654511
C	-1.848524	-0.238554	3.360671
H	-2.600726	0.245108	3.991461
H	-0.994660	1.637598	2.740055
C	1.004067	0.814551	1.118196
H	1.272043	1.721591	1.704573
H	1.961678	0.279993	0.926321
O	0.444738	1.360345	-0.050916
C	0.293776	0.436560	-1.104725
H	1.279403	0.053623	-1.461585
H	-0.282787	-0.468940	-0.802825
C	-0.390050	1.241053	-2.189274
N	-1.737436	1.127400	-2.291992
C	-2.357395	1.882714	-3.233750
C	-3.869563	1.858059	-3.254312
O	-4.348907	1.643746	-4.552143
C	-4.310755	0.309495	-4.988925
C	-5.181616	-0.605107	-4.179923
C	-6.317490	-0.133900	-3.530480
C	-7.067773	-0.988901	-2.731166
C	-6.701383	-2.324297	-2.603075
C	-7.482696	-3.250154	-1.718854
N	-6.576932	-3.918206	-0.771706
C	-7.024320	-5.010966	-0.067757
O	-8.150359	-5.484706	-0.232464
H	-5.687965	-3.470798	-0.549346
H	-8.018074	-4.004348	-2.347962
H	-8.295735	-2.716894	-1.168363
C	-5.609219	-2.813388	-3.313827
C	-4.847757	-1.954198	-4.094686
H	-3.978158	-2.347684	-4.631469
H	-5.339040	-3.872617	-3.250853
H	-7.943727	-0.599623	-2.202880
H	-6.625502	0.911051	-3.640517
H	-3.261115	-0.062543	-5.040291
H	-4.679668	0.379939	-6.036135
H	-4.255629	2.870273	-2.994757
H	-4.299407	1.159907	-2.498566
C	-1.666154	2.718983	-4.115141
C	-0.284428	2.809726	-3.998609
C	0.368137	2.078602	-3.013383
H	1.450727	2.155070	-2.880600
H	0.283931	3.460315	-4.670848
H	-2.210267	3.290307	-4.872807
H	0.823056	-1.913345	1.104836
H	-0.787124	-3.313832	2.369149
O	-2.331372	-5.223884	3.619080
H	-3.663830	-7.351641	3.344685
H	-5.351839	-8.745298	2.133358
H	-6.926735	-7.639300	0.525439

7. NMR spectroscopic data

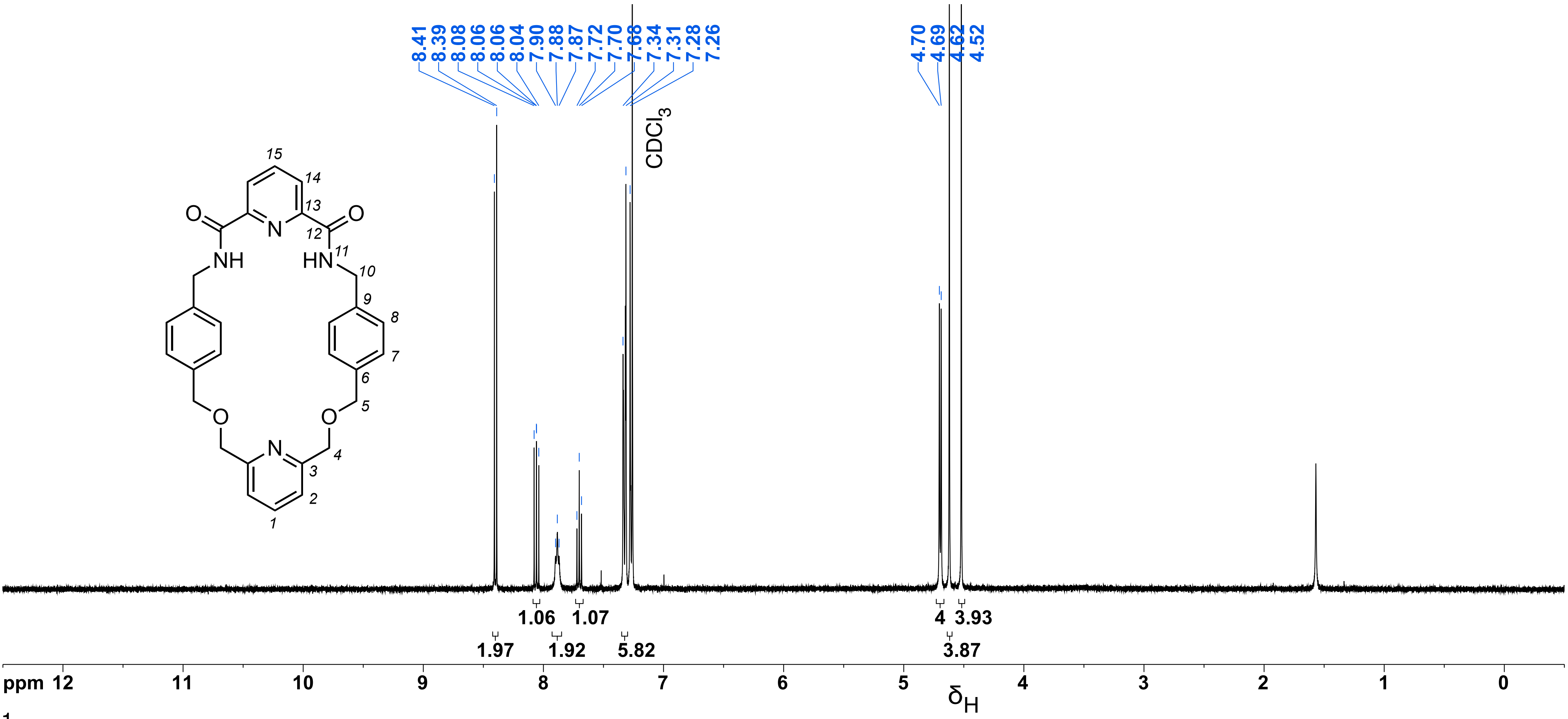

^{1}H NMR spectrum (400.1 MHz, RT, $CDCl_3$) of macrocycle **M**

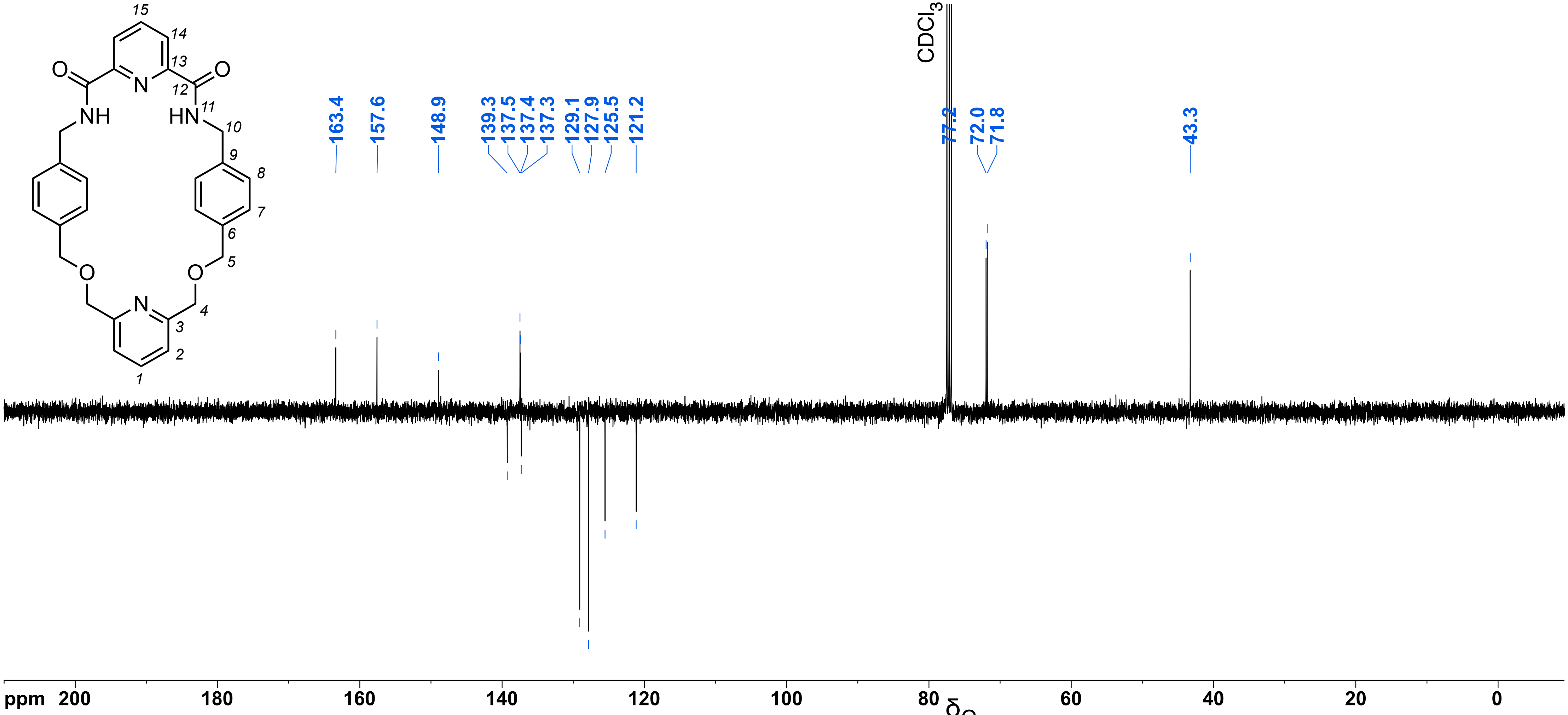

^{13}C NMR spectrum (100.6 MHz, RT, $CDCl_3$) of macrocycle **M**

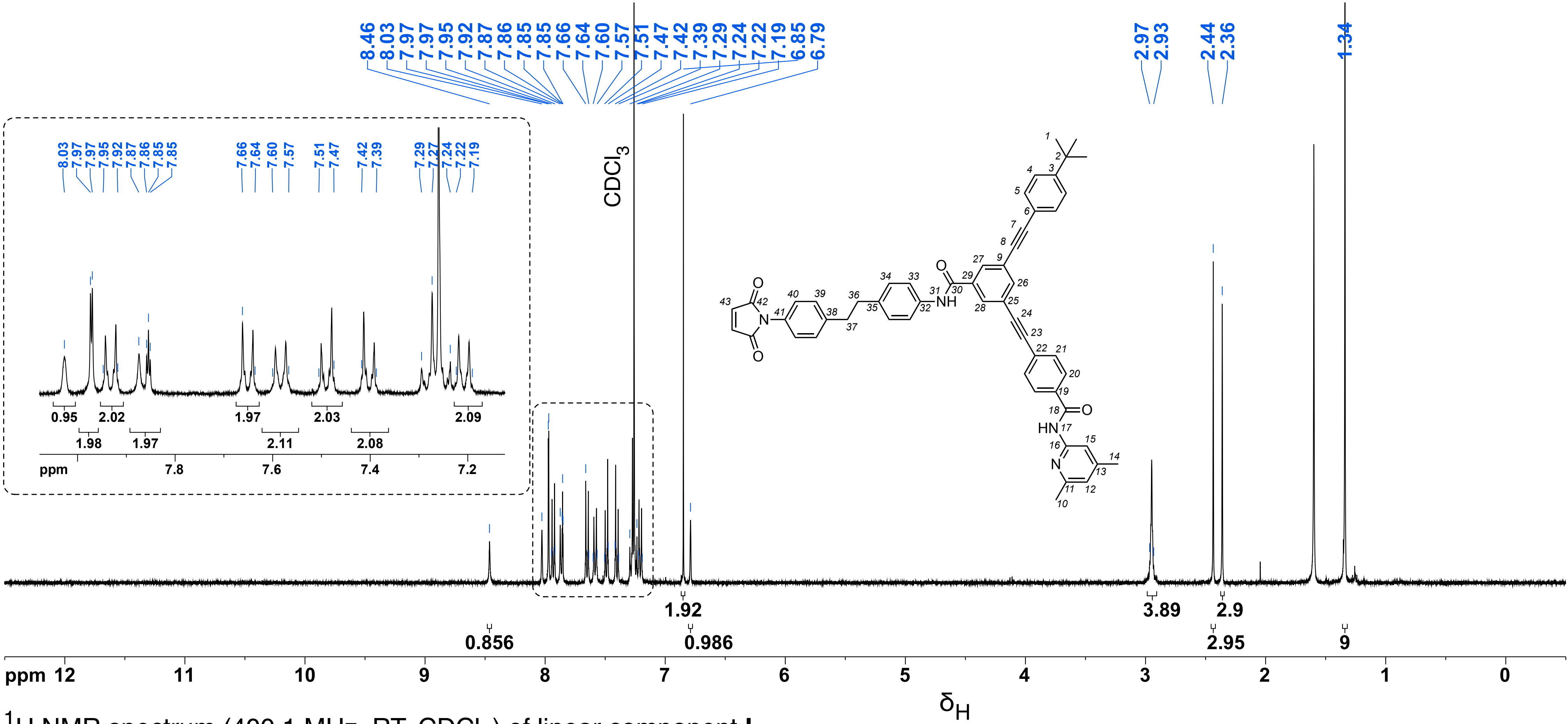

^{1}H NMR spectrum (400.1 MHz, RT, $CDCl_3$) of linear component **L**

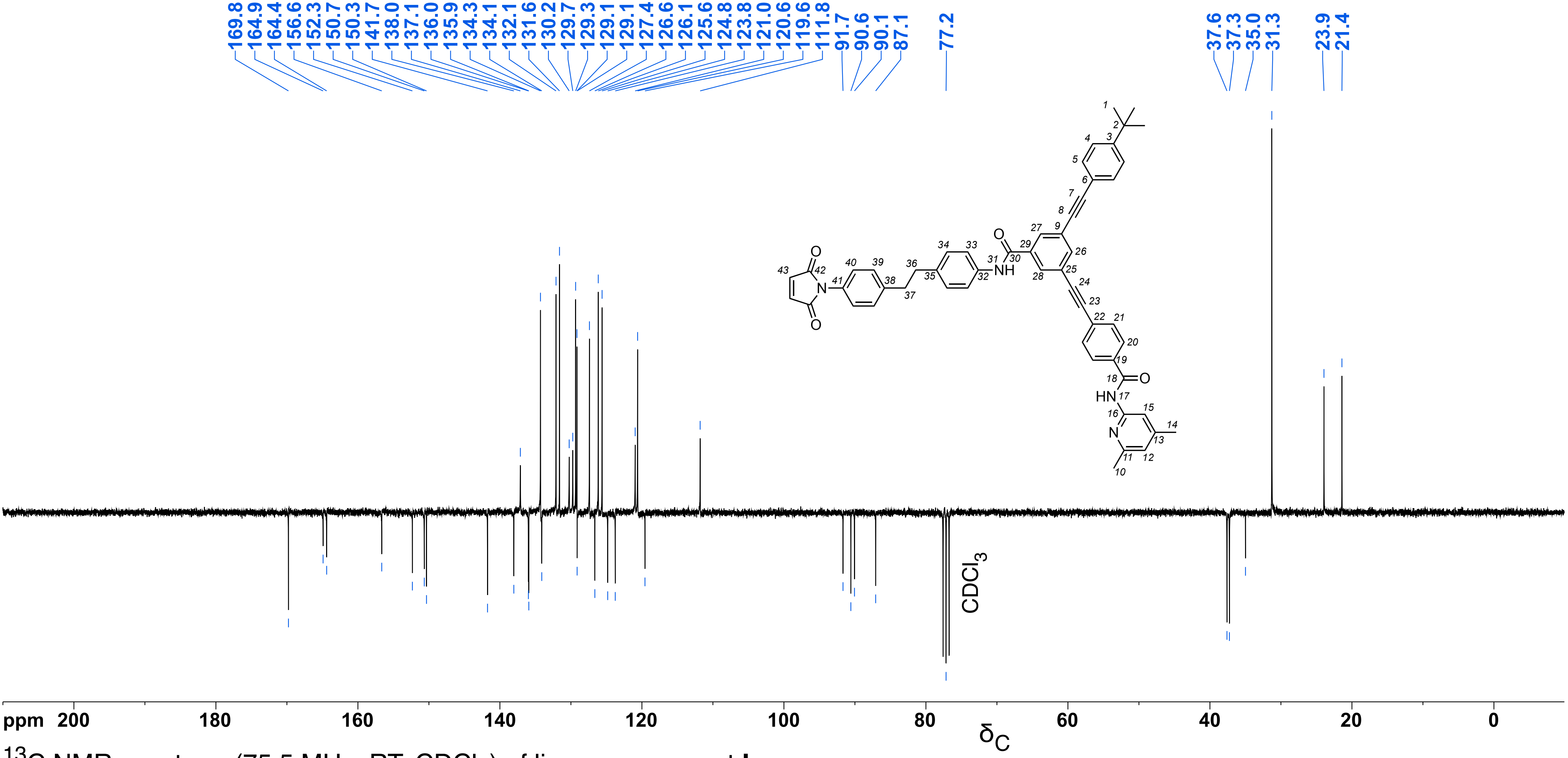

^{13}C NMR spectrum (75.5 MHz, RT, $CDCl_3$) of linear component **L**

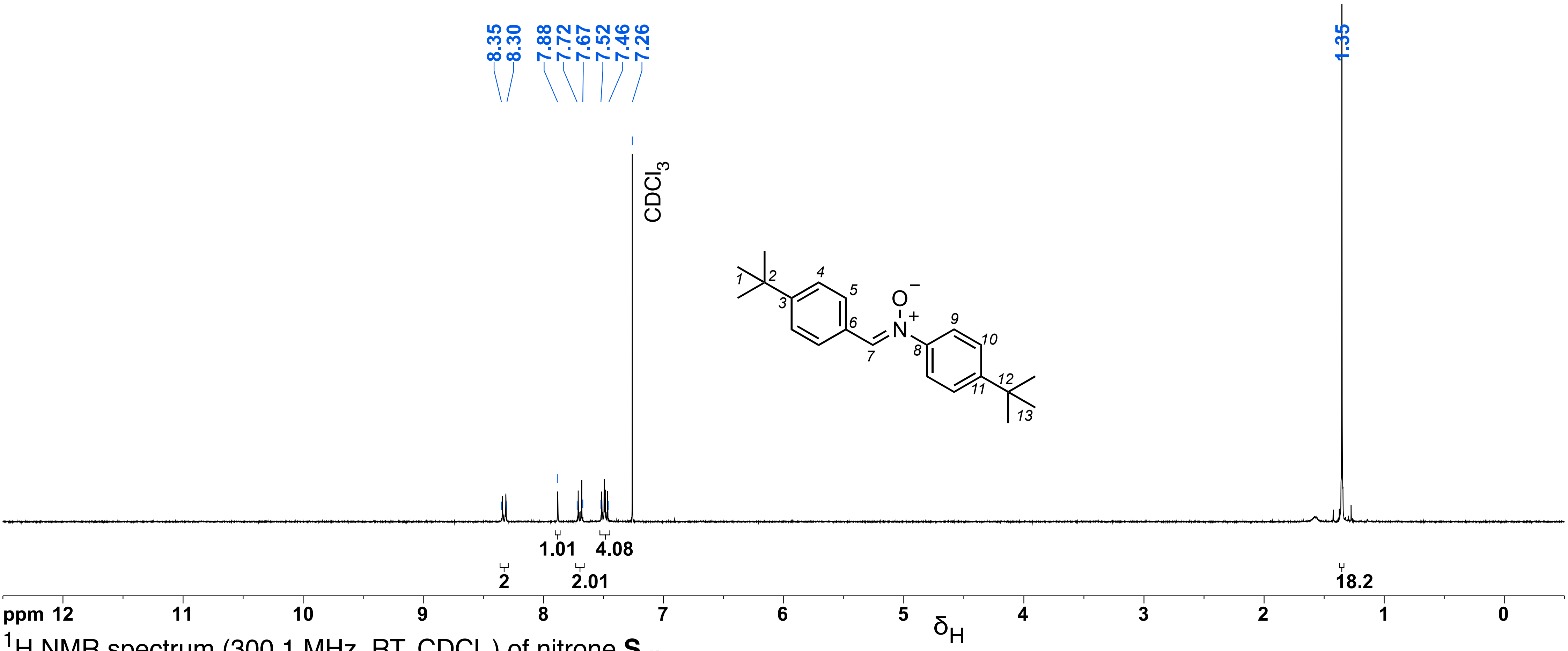

^{1}H NMR spectrum (300.1 MHz, RT, $CDCl_3$) of nitrone $\mathbf{S_{dis}}$

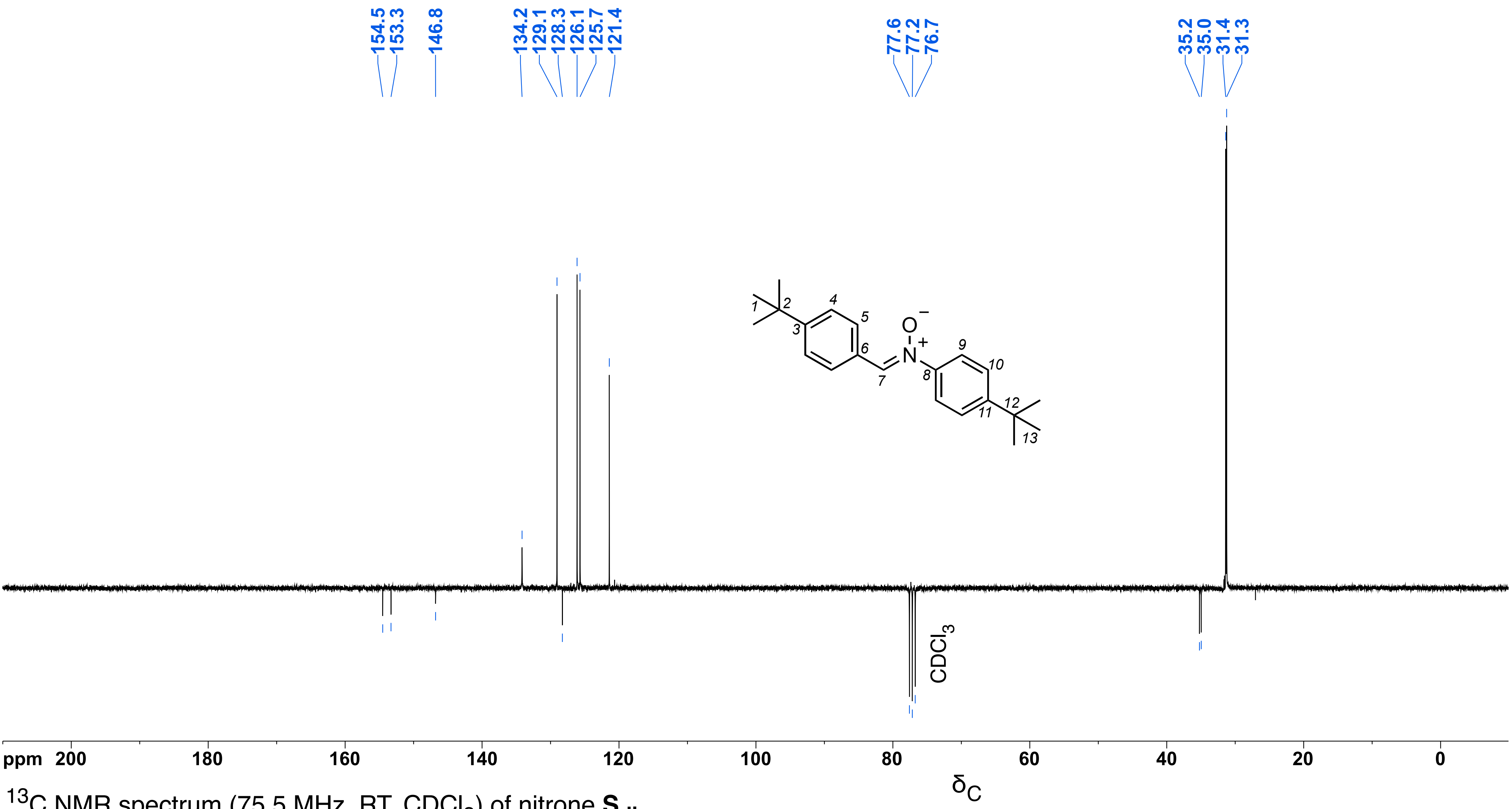

^{13}C NMR spectrum (75.5 MHz, RT, $CDCl_3$) of nitrone $\mathbf{S_{dis}}$

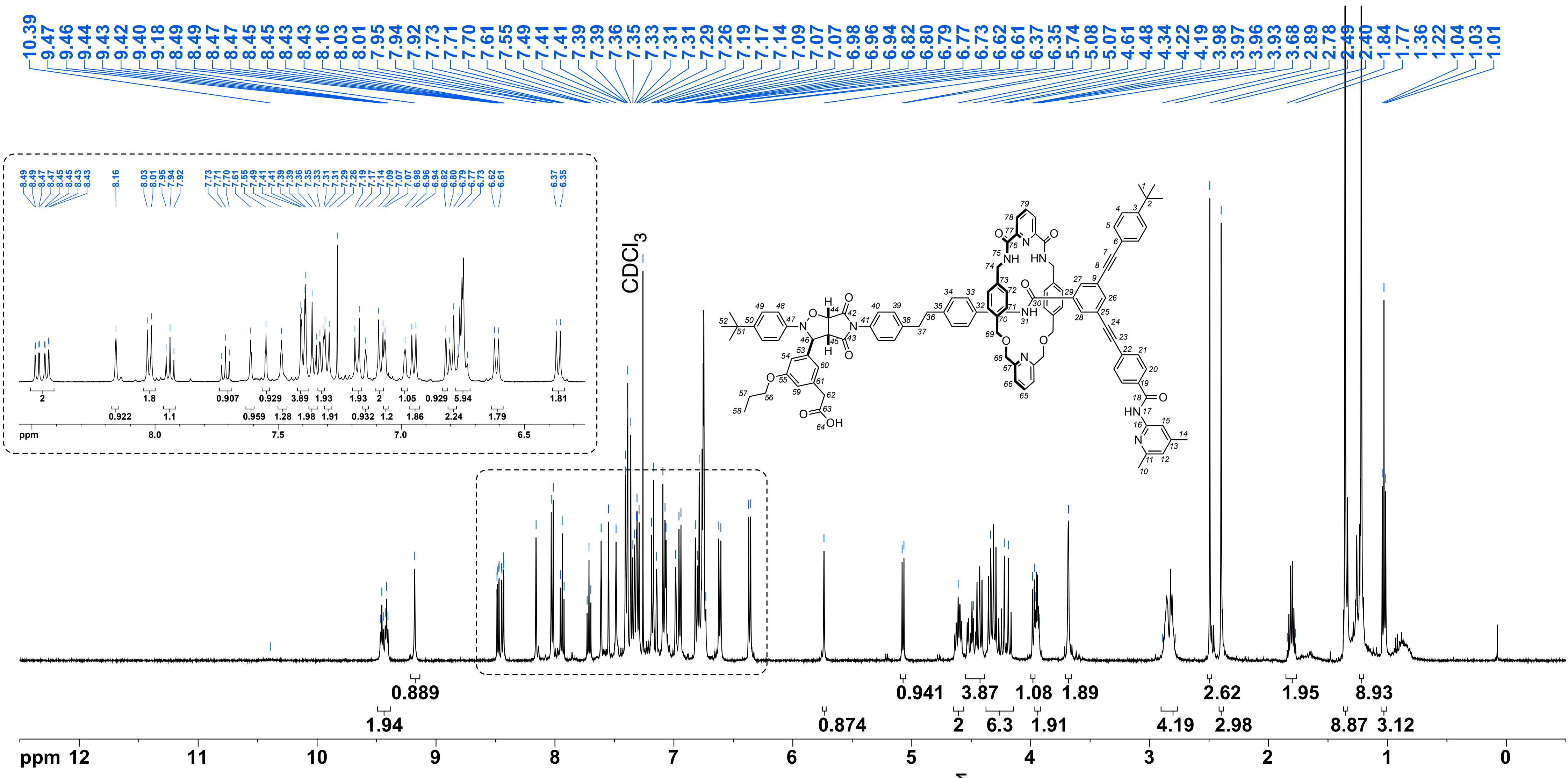

^1H NMR spectrum (499.9 MHz, RT, $CDCl_3$) of rotaxane **R**

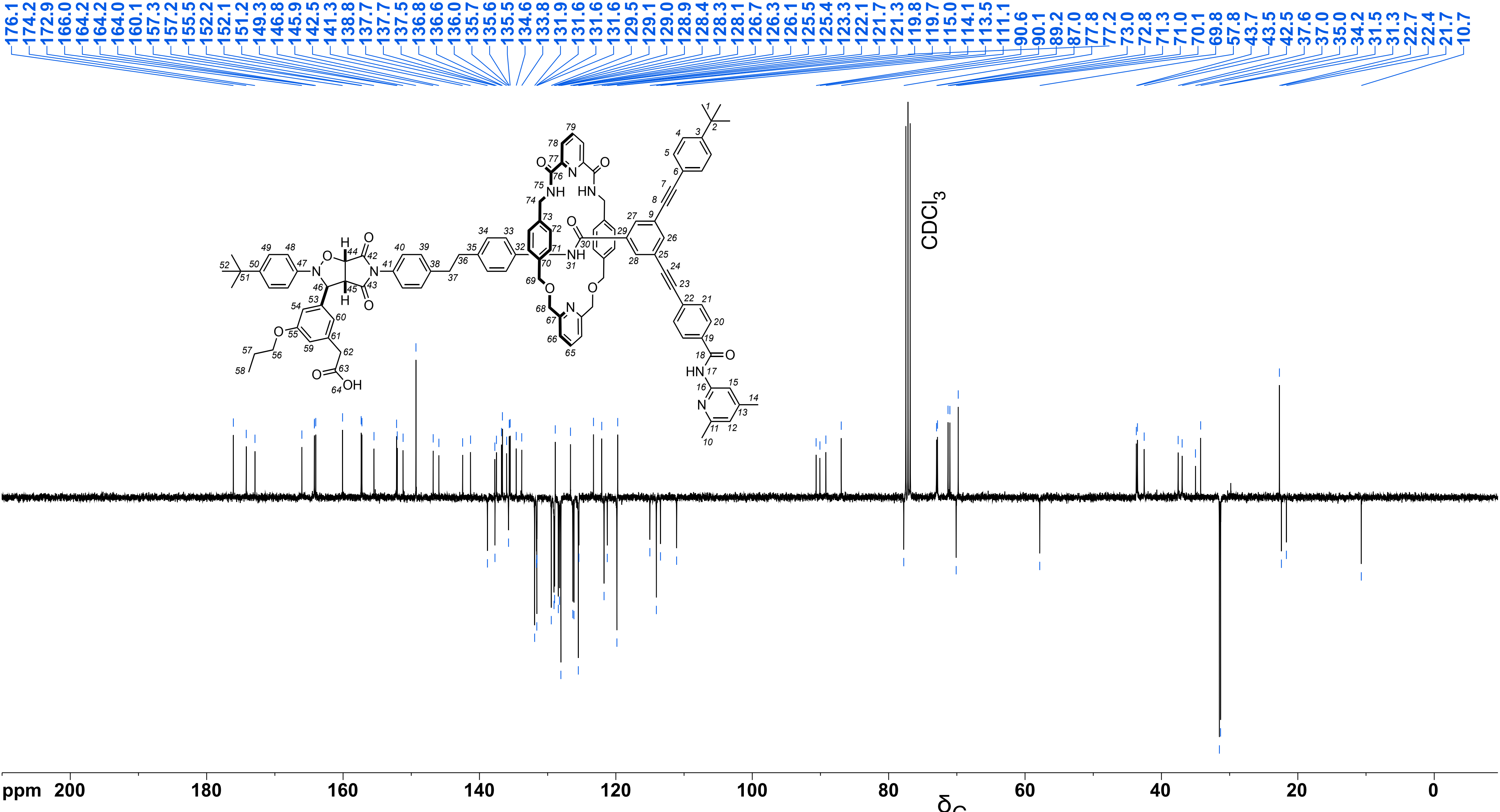

^{13}C NMR spectrum (100.6 MHz, RT, $CDCl_3$) of rotaxane **R**

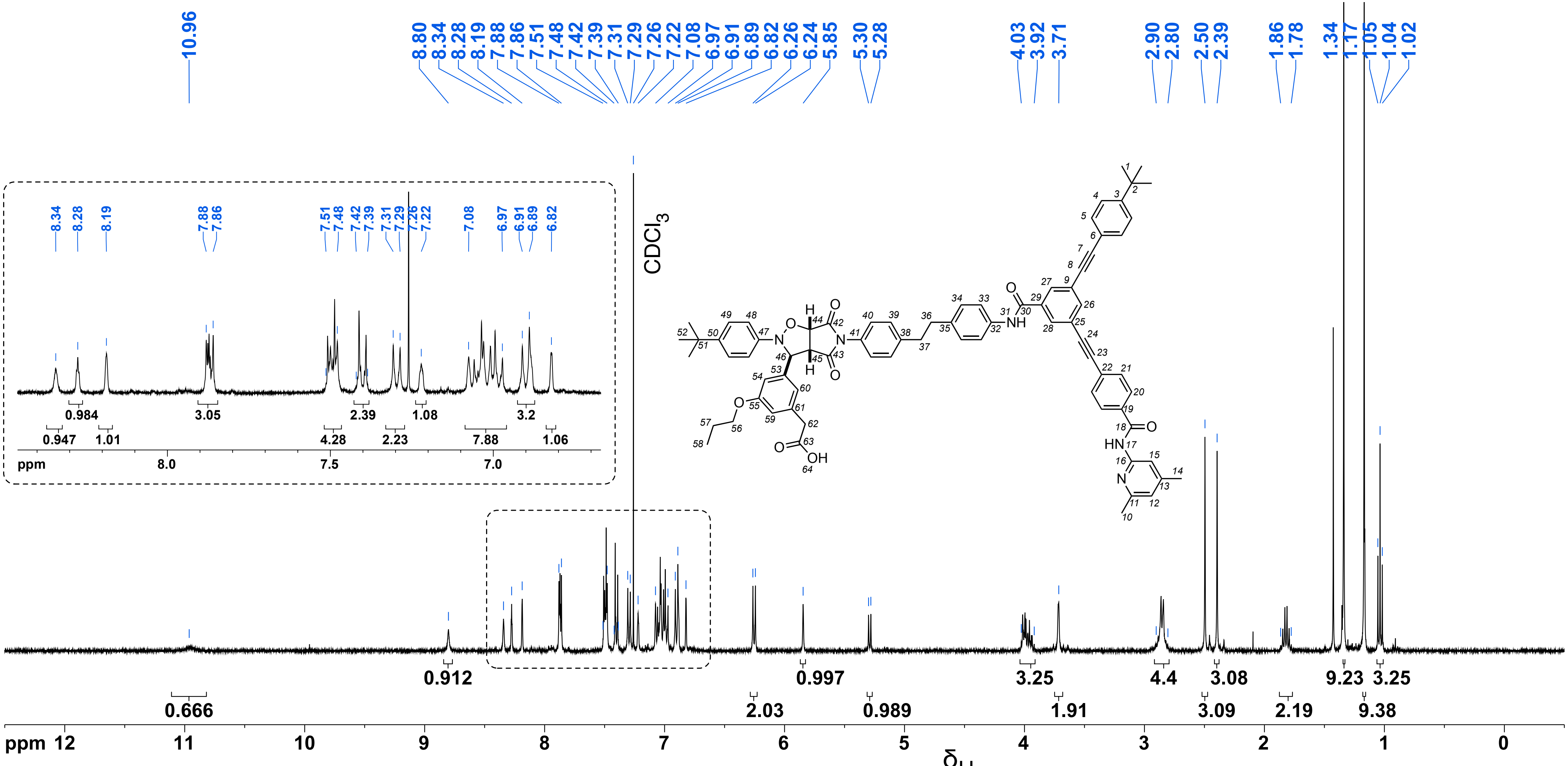

^1H NMR spectrum (400.1 MHz, RT, $CDCl_3$) of thread **T**

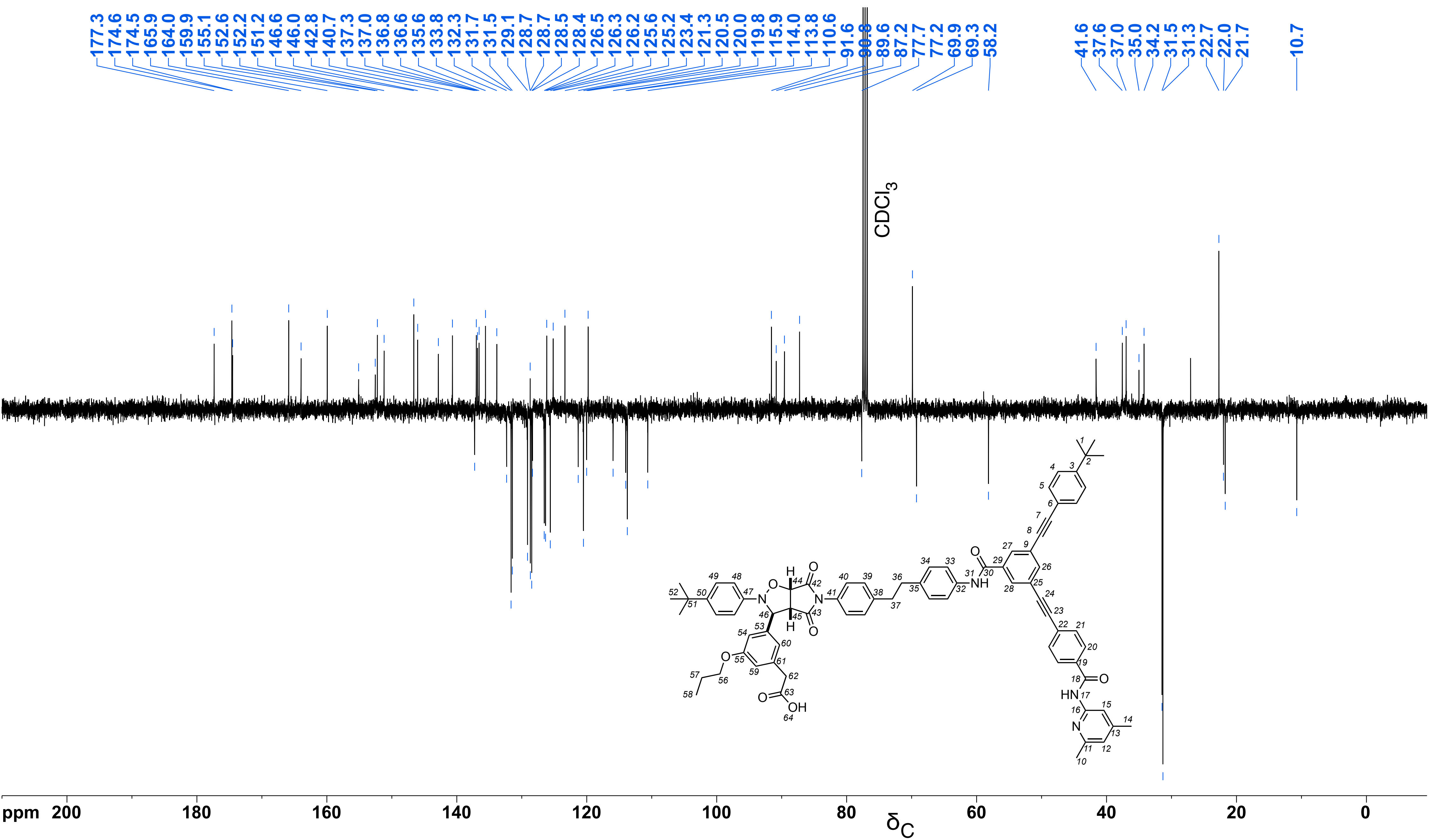

^{13}C NMR spectrum (100.6 MHz, RT, $CDCl_3$) of thread **T**
